# Changes in task-based effective connectivity in language networks following rehabilitation in post-stroke patients with aphasia

**DOI:** 10.3389/fnhum.2015.00316

**Published:** 2015-06-09

**Authors:** Swathi Kiran, Erin L. Meier, Kushal J. Kapse, Peter A. Glynn

**Affiliations:** ^1^Aphasia Research Laboratory, Speech Language and Hearing Sciences, Sargent College, Boston UniversityBoston MA, USA; ^2^Feinberg School of Medicine, Northwestern UniversityChicago IL, USA

**Keywords:** aphasia, stroke, language recovery, fMRI activations, effective connectivity, dynamic causal modeling, rehabilitation, naming

## Abstract

In this study, we examined regions in the left and right hemisphere language network that were altered in terms of the underlying neural activation and effective connectivity subsequent to language rehabilitation. Eight persons with chronic post-stroke aphasia and eight normal controls participated in the current study. Patients received a 10 week semantic feature-based rehabilitation program to improve their skills. Therapy was provided on atypical examples of one trained category while two control categories were monitored; the categories were counterbalanced across patients. In each fMRI session, two experimental tasks were conducted: (a) picture naming and (b) semantic feature verification of trained and untrained categories. Analysis of treatment effect sizes revealed that all patients showed greater improvements on the trained category relative to untrained categories. Results from this study show remarkable patterns of consistency despite the inherent variability in lesion size and activation patterns across patients. Across patients, activation that emerged as a function of rehabilitation on the trained category included bilateral IFG, bilateral SFG, LMFG, and LPCG for picture naming; and bilateral IFG, bilateral MFG, LSFG, and bilateral MTG for semantic feature verification. Analysis of effective connectivity using Dynamic Causal Modeling (DCM) indicated that LIFG was the consistently significantly modulated region after rehabilitation across participants. These results indicate that language networks in patients with aphasia resemble normal language control networks and that this similarity is accentuated by rehabilitation.

## Introduction

Most studies of language recovery have examined the recovery process in the chronic stage and have found that the recovery of language function in aphasia is a more complex process than a simple reversal of normal left hemisphere lateralization or exclusive recruitment of left perilesional and other left language areas, and likely reflects a combination of the two (Price and Crinion, 2005; Crinion and Leff, 2007; Thompson and den Ouden, 2008). Two recent cross-sectional studies highlight the complexity of the interaction. The first, a recent meta-analytic review of 12 studies by Turkeltaub et al. (2011), found that patients with aphasia showed activation in left hemisphere regions such as the left inferior frontal gyrus (IFG) and left middle temporal gyrus (MTG) that was also observed in control participants. In addition, they showed activation in new left hemisphere regions such as anterior insula and middle frontal gyrus (MFG) and homologous right hemisphere regions such as right inferior frontal gyrus (RIFG), right post central gyrus (RPCG) and right middle temporal gyrus (RMTG); none of these activation patterns were observed in control participants. According to Turkeltaub et al., patients with limited damage to the dominant/left hemisphere may demonstrate improvements due to re-engagement of spared regions and may also recruit alternate perilesional areas to subserve language recovery. In patients with large left hemisphere lesions, the engagement of the contralateral right hemisphere homologs, particularly the RIFG, is crucial to successful recovery of language. A second study (Sebastian and Kiran, 2011) examined two tasks (picture naming and semantic feature judgment) and found that while activation was observed in the LIFG in patients without lesions in the LIFG for both tasks, activation was also observed in the RIFG in all patients for the picture naming task. These studies show that undamaged regions in the left hemisphere are capable of subserving language recovery but do so in a way that is constrained by task demands and the amount of perilesional tissue available (i.e., lesion size).

The study of the neural basis of rehabilitation-induced language recovery in patients has mostly focused on whether activation in the left hemisphere or the right hemisphere is ultimately related to positive language recovery (Peck et al., 2004; Davis et al., 2006; Fridriksson et al., 2006, 2007, 2010; Vitali et al., 2007; Meinzer et al., 2008; Raboyeau et al., 2008; Crosson et al., 2009; Menke et al., 2009; Fridriksson, 2010; Marcotte and Ansaldo, 2010; Rochon et al., 2010; Heath et al., 2012). The more recent studies have highlighted the importance of left hemisphere and perilesional activation as a function of improved picture naming skills after rehabilitation (Meinzer et al., 2008; Fridriksson, 2010; Rochon et al., 2010; Marcotte et al., 2012; van Hees et al., 2014) that are consistent with Turkeltaub et al. (2011)'s suggestions about the role of the LIFG and perilesional regions in recovery. All of the above-mentioned studies have been useful in providing insight into which regions may change as a function of rehabilitation; however, these studies do not necessarily explain how these regions are modulated within a network, nor were they designed to do so.

A few studies have examined changes in network connectivity after rehabilitation (Abutalebi et al., 2009; Sarasso et al., 2010; Vitali et al., 2010). In one study, Abutalebi et al. (2009) used Dynamic Causal Modeling (DCM) to examine the effect of rehabilitation in one bilingual patient with aphasia on two different networks: the control network and the language network. The connections in these networks were measured for both languages (L1 = native language, L2 = second language) before and after therapy in L2. The authors found that rehabilitation in L2 strengthened connections within the L2 language network, but weakened connections within the L1 language network. Two other studies have also examined connectivity changes using structural equation modeling (SEM). Vitali et al. (2010) used SEM to explore rehabilitation-induced changes in connectivity among four left hemisphere language areas (IFG, MTG, insula, and IPL) and their right hemisphere homologs in two patients. For both patients, more strengthened connections were noted for trained vs. untrained items. In another study, Sarasso et al. (2010) compared left hemisphere and right hemisphere networks in four patients to a normative model at several time points throughout rehabilitation to improve articulation. They found that as rehabilitation progressed, patients' left hemisphere networks (using structural equation models) more closely resembled (i.e., were a better fit to) that of the normative model, whereas right hemisphere networks started out resembling the normal network, but progressively resembled it less. While these preliminary studies have included few subjects, they provide important preliminary evidence that improvements in behavioral rehabilitations can be reflected in terms of changes in connectivity in the language network. They also highlight the fact that there is inherent variability both within and across individuals and warrant more careful and systematic analysis of the nature of connectivity changes in patients with varying lesion and behavioral profiles.

We have also previously demonstrated that rehabilitation for naming deficits can result in positive behavioral outcomes (Kiran and Bassetto, 2008; Kiran et al., 2009, 2011; Sandberg and Kiran, 2014) and the studies reviewed above suggest that improvements after rehabilitation can be captured with neuroimaging in terms of changes in patterns of activation and inter-hemispheric shifts. The present study examined changes in patterns of BOLD signal activation and changes in connectivity as a function of rehabilitation. We implemented two behavioral tasks, picture naming and semantic feature verification, before and after rehabilitation to improve naming skills in eight patients. Both these tasks have been examined in prior studies with patients with aphasia (Postman-Caucheteux et al., 2009; van Oers et al., 2010; Sebastian and Kiran, 2011) and have a well-articulated neural framework. According to Indefrey and Levelt (2004), there are three distinct stages of word production. Selection of a word during picture naming involves regions in the left MTG, retrieval of the phonological word form involves the left posterior superior temporal gyrus (LPSTG) and middle temporal gyrus (LMTG), and finally, planning of the phonological form (i.e., syllabification) involves the left posterior inferior frontal gyrus (LIFG). Likewise, Binder and colleagues (Binder et al., 2009; Binder and Desai, 2011) have proposed a neural framework for semantic processing that includes modality-specific input to temporal and parietal regions that process modal representations of semantic knowledge. Also, frontal regions including dorsomedial and inferior prefrontal regions are involved in the goal directed activation of information from the temporal regions.

Since the rehabilitation was focused on strengthening semantic representations through feature verification to improve picture naming, the fMRI tasks focused on the outcome of therapy (picture naming which requires semantic and phonological access) as well as the cognitive mechanism targeted in therapy (semantic processing during the semantic feature verification). Thus, in addition to the fact that both the picture naming and semantic feature tasks have been well-examined in fMRI experiments across both patients with aphasia and normal controls, these tasks are well-tailored to the behavioral language rehabilitation implemented in the study and compensate for the modest number of participants by providing within-subject replication across similar tasks. The following were the research aims and hypotheses proposed in the present study:

1. To examine behavioral changes in patients with aphasia who receive a semantic feature-based naming therapy. Our previous work has shown that a theory based rehabilitation aimed at improving lexical retrieval for atypical examples results in improvement of trained items as well as generalization to untrained items within the same category (Kiran and Thompson, 2003; Kiran, 2008; Kiran and Bassetto, 2008; Kiran and Johnson, 2008; Kiran et al., 2011) leading to the Complexity Account of Treatment Efficacy (Kiran, 2007; Thompson, 2007). In the present study, we implement this well-established protocol by training atypical examples from different semantic categories across patients, with the expectation that the semantic feature-based treatment will result in improved naming of the trained items as well as generalized naming of untrained items within the same category. The goal of this study was not to examine the patterns of generalization; therefore, although they were collected, these data will not be reported.
2. To explore changes in activation on two tasks: picture naming and semantic feature matching. Based on previous studies examining task-based activation for picture naming and semantic feature matching and data collected from healthy normal controls reported in this paper, we expect to see activation in the language network that encompasses regions including temporal regions (ITG, MTG, STG), inferior parietal regions [supramarginal gyrus (SMG), angular gyrus (AG)], precentral gyrus (PCG) and the dorsolateral (MFG, SFG), and inferior frontal regions (IFG), in patients with aphasia who have residual tissue in these regions. Furthermore, we expect changes in activation in these regions in patients who show improvements in language function after rehabilitation.
3. To explore changes in effective connectivity using DCM. Given the current robust evidence for regions involved in the two tasks examined in this experiment, DCM is the ideal methodology as it is hypothesis-driven and allows researchers to examine whether rehabilitation changes the nature and strength of connectivity between language regions in patients. Importantly, DCM has been used to examine the nature of the damaged or reorganized network in clinical populations (Sonty et al., 2007; Grefkes et al., 2008; Abutalebi et al., 2009; Rehme et al., 2011; Campo et al., 2013; Kahan and Foltynie, 2013); however, there is only one case study examining DCM to measure changes in connectivity as a function of rehabilitation (Abutalebi et al., 2009). Notably, we employed this method at the single participant level due to the inherent variability across our participants in terms of lesion sites and responsiveness to rehabilitation. Therefore, we expected to see differences in networks that changed as a function of rehabilitation within patients, but also expected to see certain regions (e.g., LIFG) that were modulated as a function of treatment across participants.

## Materials and methods

### Participants with aphasia

**Figure 1:**
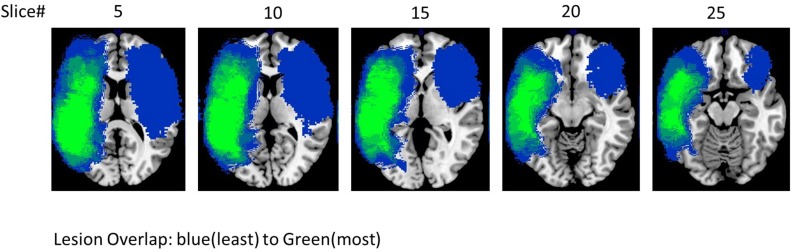
**Lesion overlap of all the eight patients**.

Eight participants with aphasia (mean age = 58 years; seven male), all of whom had a single stroke, participated in the study. All participants had infarcts in the left hemisphere with the exception of #5 who had a stroke in the right hemisphere^1^. All patients were given a battery of standardized language tests, including the Western Aphasia Battery-Revised WAB-R (Kertesz, 2007) to establish the type and severity of aphasia, the Boston Naming Test (BNT) (Goodglass et al., 1983; Kaplan et al., 2001) to determine confrontation naming ability, the Pyramids and Palm Trees (PAPT) (Howard and Patterson, 1992) to determine overall soundness of the semantic system, and the Cognitive Linguistic Quick Test (CLQT) (Helm-Estabrooks, 2001) to determine the relative contribution of cognitive deficits such as attention and visuo-spatial skills to language dysfunction. Due to the linguistic nature of the tasks included in the language and memory subscales of the CLQT, these scores are not included in the table; it should be noted, however, that language and memory scores (as well as other cognitive domain scores) do contribute to the CLQT composite score. As can be seen in Table 1, patients presented with varying levels of language impairment ranging from 48 to 97.2 on the WAB AQ, and 6.6 to 85% on the BNT. Of note, lesion volume did not correlate with language impairment either on the WAB (*r* = −0.4, *p* = *ns*) or BNT scores (*r* = 0.07, *p* = *ns*) (see Figure 1 for a lesion overlap map).

**Table 1:** **Demographic information for participants in the study that include age, months post onset (MPO), Western Aphasia Battery Aphasia Quotient (WAB AQ), Boston Naming Test (BNT), Pyramids And Palm Trees (PAPT, 3 pictures test), Cognitive Linguistic Quick Test (CLQT), and overall lesion volume in cc**.

**Patient #**	**(in years)**	**MPO**	**Overall lesion volume (in cc)**	**WAB AQ**	**BNT score (%)**	**PAPT (three pictures)**	**CLQT (Composite Severity)**	**CLQT (Attention)**	**CLQT (Visuospatial Skills)**
5	53	107	287.17	75.4	83	96.1	Mild	WNL	WNL
11	59	143	235.03	71.2	60	94.2	Mild	WNL	WNL
15	59	15	168.46	85.1	85	94.2	Mild	WNL	WNL
32	51	87	247.1	48	6.6	88.4	Mild	Mild	WNL
33	65	49	114.83	49.6	15	96.1	Mild	Mild	WNL
62	49	157	431.63	58.2	58.1	96.1	Moderate	Mild	Mild
93	66	24	24.21	97.2	65.5	92.3	Mild	WNL	WNL
115	63	98	208.05	53.4	15	96.1	Mild	Mild	WNL

*The CLQT composite severity computes a profile by measuring attention, memory, executive functions, language, visuo-spatial skills, and clock drawing abilities. WNL, Within normal limits*.

In addition, six tests assessing semantic and phonological processing were administered to examine the nature of phonological processing, semantic processing and naming. While performance varied across participants, semantic processing scores were higher than phonological processing scores in general (see Table 2). The only behavioral criteria for inclusion into the experiment was the presence of a naming impairment (<70% accuracy) on a set of pre-determined 96 pictures that varied by their category typicality, see Table 2 for details. Of note, WAB AQ correlated with the average naming skills for atypical examples (*r* = 0.65, *p* < 0.05) indicating that the naming impairment was consistent with their overall language impairment profile. All participants gave consent according to BU IRB protocol.

**Table 2:** **Performance on behavioral measures prior to treatment, including phonological processing tasks (rhyme judgment, syllable judgment, and phoneme judgment), semantic processing tasks (coordinate task, superordinate task, semantic feature), and picture naming (atypical and typical examples) collapsed across categories**.

**Patient #**	**Rhyme judgment (%)**	**Syllable judgment (%)**	**Phoneme judgment (%)**	**Coordinate judgment (%)**	**Superordinate judgment (%)**	**Semantic feature judgment (%)**	**Average naming typical (%)**	**Average naming Atypical (%)**
5	100	87.00	67.00	ND	ND	80.00	68.94	56.82
11	ND	ND	ND	ND	ND	ND	70.83	61.11
15	78.75	78.75	65.00	98.75	97.50	88.75	65.74	67.59
32	51.25	51.25	47.50	81.25	82.50	87.50	30.00	26.19
33	45.00	45.00	55.00	97.50	90.00	88.75	30.83	18.33
93	77.50	65.00	57.50	87.50	95.00	86.25	68.52	59.26
62	55.00	52.50	46.25	62.50	80.00	80.00	41.67	47.22
115	53.75	66.25	ND	ND	ND	85.00	25.00	31.48

*ND, no data*.

### Control participants

Eight control participants (mean age = 57.5 years, four males) with no history of brain damage were recruited to obtain normative data on task-specific activation for the two language tasks. Exclusionary criteria included neurological disorders such as stroke, transient ischemic attacks, Parkinson's disease, Alzheimer's disease, psychological illness, learning disability, seizures, and attention deficit disorders. The controls did not receive language therapy and were scanned at only one time point. Half the controls were scanned as part of a different ongoing experiment, the details of which are provided in Supplementary Table 1. All participants gave consent according to BU IRB protocol.

### Stimuli

Five semantic categories (*birds, vegetables, furniture, clothing*, and *musical instruments*) were used to study naming and semantic processing during the rehabilitation tasks across the eight patients. Twenty-four items were selected for each category that comprised typical and atypical examples (e.g., *bird: typical–robin, atypical–ostrich; vegetables: typical–spinach, atypical–mushrooms, clothing: typical–sweater, atypical–apron; furniture: typical–dresser, atypical–chandelier; musical instruments: typical–violin, atypical–bagpipe)*. Patients were exposed to three of the five categories: a trained category (probed weekly), an assessed category (only tested before and after rehabilitation) and a monitored category (probed at the same frequency as the trained category). The assignment of trained, untrained, and monitored categories was counterbalanced across participants and constrained by their initial naming accuracy. Hence if the patient named more than 70% of the items in the category at baseline, that category was eliminated from potential set of stimuli for that patient. Each category contained 12 typical and 12 atypical examples that were selected from our previous rehabilitation studies (Kiran and Thompson, 2003; Kiran, 2008). All stimuli were concrete nouns balanced for length, frequency of occurrence (CELEX, Vanderwouden, 1990), familiarity, and concreteness (http://websites.psychology.uwa.edu.au/school/MRCDatabase/uwa_mrc.htm) (Coltheart, 1981). Each patient was trained on 12 examples within each category.

### Semantic features employed during treatment

The typicality treatment employed analysis of semantic attributes of concepts. Semantic features were selected from our previous studies (Kiran and Thompson, 2003; Kiran, 2008). Each category contained 40 semantic features, each of which was applicable to at least two items within the category, and each item within the category could be assigned at least six features. Equal numbers of distractor features were included. Semantic features were controlled for whether they were defining or characteristic of the category and for type of information conveyed; i.e., equal number of physical, functional, and contextual features.

### Treatment and monitoring protocol

Confrontation picture naming was tested during baseline sessions, and then treatment was applied to one set of items within a category. In each session, semantic attributes of the target category were presented to the patient in order to strengthen the semantic representation of that specific category (Kiran and Bassetto, 2008). Participants practiced the following steps for each of the trained items: (1) analysis and selection of six semantic features of the target item, (2) answering 15 Y/N questions of which five belong to the target example (e.g., “has wings”), five belong to the category but not the target example (e.g., “flies”), five that do not belong to the target category (e.g., “worn on body”), and (3) naming the target picture (e.g., ostrich).

Throughout rehabilitation, weekly naming probes were administered to assess naming of the trained and untrained items within the trained category and untrained categories. The assessed category was only probed during the pre- and post-rehabilitation sessions. Rehabilitation was terminated when each patient named at least 10/12 (80% accuracy or higher) items accurately across two consecutive sessions. Subsequently, three post-rehabilitation naming probes, using the same procedures as the baseline and rehabilitation probes, were administered to calculate the efficacy of rehabilitation. Effect sizes^2^ and percent change were calculated to determine the degree of change in pre-post rehabilitation performance (Busk and Serlin, 1992).

### Neuroimaging experiment and design procedures

Both patients and control participants participated in the fMRI experiment. As noted before, the controls completed the fMRI experiment once in order to identify regions of activation that are normally engaged for the two tasks. All patients completed two fMRI scans, one during the baseline testing phase (pre-treatment scan) and one during the post-rehabilitation phase (post-treatment scan).

### fMRI task design

**Figure 2:**
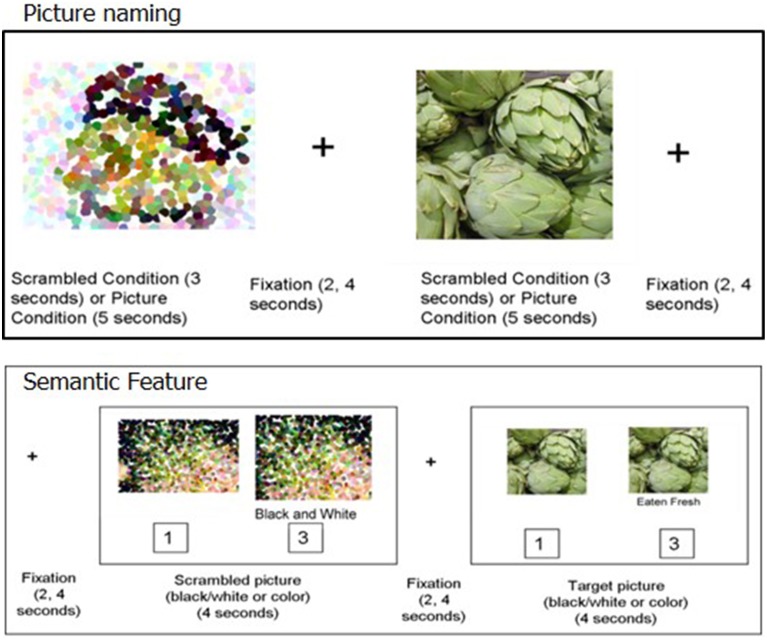
**Picture naming (top) and Semantic feature task (bottom)**.

For both picture naming and semantic feature tasks (shown in Figure 2), an event-related design using randomized inter-stimulus intervals (ISIs) was implemented using E-Prime 2.0 (Psychology Software Tools, Inc.). For both tasks, ISIs were jittered between 2 and 4 s (Birn et al., 2004). In this design, when averaged, the jittered ISI accounts for speaker-related brief motion artifacts and has been successfully implemented for several overt-naming tasks that do not use sparse sampling (Birn et al., 2004; Meltzer et al., 2009; Menke et al., 2009; Postman-Caucheteux et al., 2009).

For the picture naming task, 80 stimuli included items from the trained category and the untrained- assessed category (except for P1 and P2). Four different runs of the picture naming task were used, each containing 20 experimental stimuli (pictures to name) and 20 control stimuli (scrambled pictures). Two runs consisted of stimuli from the trained category and the other two runs consisted of stimuli from the untrained categories. Participants were required to name each picture aloud or say “SKIP” for pictures they could not name. The control stimuli were pixelated, scrambled versions of the experimental stimuli. Participants were required to say “SKIP” for each control item. The experimental pictures were presented for 5 s while the scrambled control stimuli were presented for 3 s.

In the semantic feature verification task, 80 stimuli were evenly distributed across the **four** categories. In both the experimental and control conditions, each trial consisted of a picture prime (presented for **1** s) followed by the target stimulus (presented for **4** s). Half of these prime stimuli and their corresponding target stimuli were presented in black and white and the other half were presented in color. The target stimulus was the prime picture repeated with a written phrase below. This design was used to provide enough time for patients with aphasia to process the visual attributes of the picture prior to making a decision. In the experimental condition, the written phrase was a semantic feature, and participants indicated by button press whether the written semantic feature applied to the pictured item. The control stimuli contained a scrambled picture (presented in either black-and-white or color) and a statement regarding the color of the scrambled picture (either “black and white” or “color”). Participants indicated by button press whether the statement regarding the color of the picture was true or false (see Figure 2 for details).

### fMRI data acquisition

Magnetic resonance images were acquired at Boston University Center for Biomedical Imaging on a 3 Tesla Philips Acheiva MRI scanner. High-resolution T1-weighted images were acquired with the following parameters: 140 sagittal slices, 1 mm^3^ voxels, 240 × 240 matrix, FOV = 240 mm, flip angle = 8, fold-over direction = AP, TR = 8.2 ms, TE = 3.8 ms. Blood-oxygen-level-dependent (BOLD) sensitive functional images were collected using the following parameters: 31 axial slices, 3 mm thick, 0.3 mm interslice gap, 80 × 78 matrix, FOV = 240 mm, flip angle = 90, fold-over direction = AP, TR = 2000 ms, TE = 35 ms. Picture naming responses were recorded in software OptiMRI 2.4 (dual channel) with live noise cancelation. Semantic feature responses were recorded using a left hand button response box for both groups of participants.

### fMRI data analysis

#### Preprocessing

Preprocessing was performed to correct for slice timing differences and movement, and to remove slow baseline drifts. Data were analyzed using SPM8 software (Wellcome Trust Centre for Neuroimaging). Slice timing correction was applied with reference to the middle slice. Structural scans were coregistered to a mean functional image obtained from realignment performed on functional scans for motion correction. For each patient, a lesion map was drawn on their T1 image using MRIcron (http://www.cabiatl.com/mricro/) (see Table 1). Unified segmentation was performed based on coregistered structural images into gray matter, white matter and CSF. A masking image was provided during segmentation so that the regions containing a value of zero would not contribute to the analysis when estimating the segmentation parameters (Brett et al., 2001; Meinzer et al., 2013). Structural and functional images were spatially normalized to the default MNI template in SPM8. Slow baseline drifts were filtered out using a high-pass filter with a cutoff of 1/128 s. Spatial smoothing of functional data was not performed to minimize the loss of specific activations that can occur due to smoothing (Meinzer et al., 2013).

Several steps were performed in the pre-processing of the data to address potential movement-related artifacts on the picture naming task. In addition to slice timing and co-registration, motion correction in SPM8 (Realign) was utilized. All fMRI images/volumes were registered to mean slice. Also, the realignment parameters (motion correction parameters) were used as regressors in the first level GLM analysis. In addition, for specific individuals (#5, 11, 15, 62), volumes with large variations (>0.05 mm) in scan-to-scan motion were repaired via linear interpolation using the ArtRepair toolbox in SPM8 (Mazaika et al., 2009).

#### First level analysis

First level analysis was performed based on the General Linear Model (GLM) in SPM8. Task timings (stimulus onsets and durations) were convolved with the canonical hemodynamic response function (HRF) and its temporal derivative. Conditions included pictures, scrambled pictures, and fixations, and were modeled in the GLM separately for each scan. Motion parameters were included in the model as regressors. Serial correlations were accounted for using an AR (1) model. The model was estimated using a restricted maximum likelihood approach (ReML). For the patients, pre-rehabilitation and post-rehabilitation scan activation maps were calculated based on *t*-test contrasts for the pictures—scrambled contrast for the trained and untrained category separately. The main contrast of interest was [post-rehabilitation (picture-scrambled)]-[pre-rehabilitation (picture-scrambled)] for each task. Activations maps were thresholded at a family wise error threshold (FWE) < 0.05. Uncorrected *p* < 0.001 activations were examined when FWE thresholds were not significant. Coordinates for activated voxels were entered into the Anatomy toolbox, v.17, to obtain the label for each active region. For normal controls, all four runs (two from each category) were combined into one GLM for each task as no treatment was provided.

#### Effective connectivity analysis

Effective connectivity analysis was applied using the DCM toolbox in SPM8. DCM uses differential equations to model inter-regional interactions to infer their directionality and context-dependent modulations (Seghier et al., 2010; Stephan et al., 2010). It is a hypothesis-driven modeling method testing for effect of task on and between regions. The constructed models calibrate the neuronal activity into hemodynamic responses and estimate the parameters based on observed fMRI signal. To apply DCM, a set of models is defined with regions and their intrinsic connections in the form of a matrix labeled DCM-A. This is followed by applying task effect modulation(s) to connection(s) (DCM-B) and regions(s) (DCM-C). The Bayesian estimation provides estimated parameters for each model and its subsequent connection and region as Ep.A, Ep.B, and Ep.C. The averaging tools in DCM provide inference either at the model level by computing the Bayesian Model Average (BMA) in each session, or at the connection level by computing the Bayesian Parameter Average (BPA) across all sessions. As will be discussed in detail below, in this study, only connection parameters (Ep.B and Ep.C) from the BPA were used to investigate patterns of connectivity for normal controls and changes in connectivity as a function of rehabilitation for patients.

#### VOI selection and model specification

All normal healthy controls showed overlapping activation in language areas for each task; thus, a common model space was constructed for this group (Seghier et al., 2010). As shown in **Tables 4, 5**, regions which were active across all controls for the picture > scrambled contrast in each task were selected as potential voxels of interest (VOIs). This resulted in 12 VOIs for the picture naming task and 12 VOIs for the semantic feature task. Within an active cluster, the voxel with the highest *T*-value was selected. Subject-specific eigenvariates were extracted as spheres of 5 mm around the MNI coordinate of the peak voxel and adjusted for the effect of interest.

Because the patients presented with varying sizes and sites of lesion and corresponding fMRI activation patterns, extraction of a common set of VOIs across patients was not possible. Thus, for each patient, a common active voxel of interest (VOI) present at both the pre- and post-rehabilitation scan (at *p* < 0.001 uncorrected) was extracted for each of the two tasks using the same procedure as that described for normal controls. All DCM models were deterministic, bilinear, two-state with mean-centered inputs. The DCM models were set up based on guidelines from previous studies (Abutalebi et al., 2009; Seghier et al., 2010; Rehme et al., 2011; Kahan and Foltynie, 2013). Across both the groups, within-hemisphere connections were defined for all the regions while between-hemisphere connections were defined for homologous regions only.

#### Extraction of Bayesian Parameter Average (BPA) values

Bayesian Model Selection (BMS) with random-effects (rfx) was initially performed on each model space to find the best fit model. Due to the variability in activation within patients, the best fit model was not uniform across patients and, hence, was not consequently pursued for controls or patients. Instead, for both groups, BPA was computed to investigate connectivity parameters for each connection (Ep.B) and region (Ep.C). For controls, these connectivity parameters were plotted to create a normal language network for each task that then served as a reference framework to evaluate the patient rehabilitated networks. For patients, BPA parameters were computed for trained and untrained categories separately. These subject-specific parameter estimates were used for second-level analysis (rANOVA, *t*-tests) to understand changes after rehabilitation (Stephan et al., 2010).

## Results

The results of the study are organized into three sections. First, we discuss the behavioral rehabilitation results, followed by fMRI activation and connectivity patterns for the controls, and lastly by fMRI activation and connectivity patterns for the patients.

### Rehabilitation results

As can be seen in Table 3, all patients improved after rehabilitation, as noted by effect sizes for the trained examples irrespective of the category trained. Therefore, all participants show medium to large effect sizes for the trained examples (Beeson and Robey, 2006). One-way ANOVAs on the average effect size and percent change on the trained category, untrained monitored category (monitored every week) and the untrained assessed category (before and after rehabilitation), showed a significant effect of category on effect size [*F*_(2, 24)_ = 17.3, *p* < 0.0001] and percent change [*F*_(2, 24)_ = 29.5, *p* < 0.0001]; *post-hoc* tests show a significantly larger effect size and percent change for the trained category relative to the untrained assessed category (*p* < 0.0001) and the untrained monitored category (*p* < 0.00001). Therefore, a clear effect of rehabilitation on the trained category was observed.

**Table 3:** **Effect sizes (ES) and percent changes for the trained categories across participants compared with untrained but weekly monitored category and untrained but assessed category as a function of rehabilitation**.

**Patient #**	**Trained set**			**Untrained probe set**			**Untrained assessed set**		
	**Category**	**ES**	**Percent change**	**Category**	**ES**	**Percent change**	**Category**	**ES**	**Percent change**
05	Vegetables	5.02	40%	Birds	2.18	14%	Musical instruments	0.74	8%
11	Birds	6.24	39%	Vegetables	1.50	13%	Musical instruments	−0.34	0%
15	Birds	4.53	22%	Vegetables	0.74	15%	Furniture	1.16	13%
32	Vegetables	3.29	20%	Birds	1.09	10%	Furniture	0.45	5%
33	Clothing	8.94	49%	Birds	1.56	19%	Furniture	0.08	0%
62	Clothing	2.68	24%	Vegetables	1.59	11%	Furniture	0.52	7%
93	Vegetables	2.84	36%	Birds	0.97	10%	Clothing	0.94	10%
115	Birds	9.51	46%	Furniture	2.90	18%	Vegetables	2.69	15%

### Activation and connectivity results for controls

#### fMRI activation results

**Figure 3:**
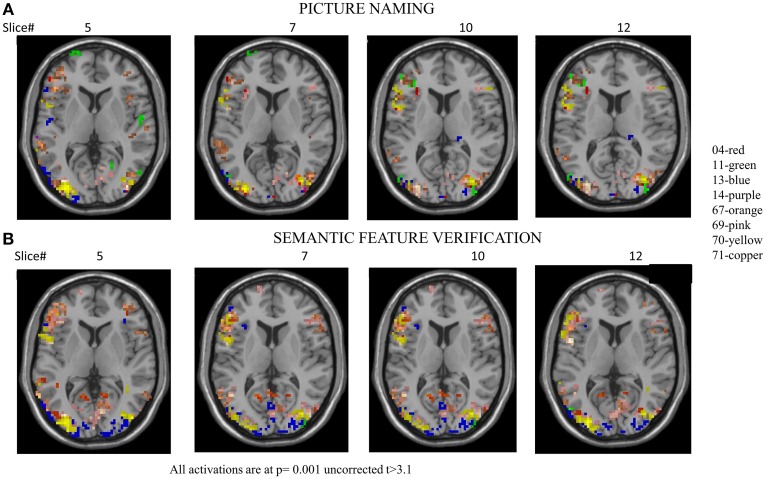
**(A)** Overlap activation maps of the eight healthy controls for picture naming task. **(B)** Overlap activation maps of the eight healthy controls for the semantic feature verification task. The voxels are at threshold based on *T* > 3.10, *p* = 0.001. See Tables 4, 5 for individual thresholded activation.

Activation patterns for normal controls are provided for each individual control across regions that are broadly involved in language processing. Table 4 and Figure 3A show activation for each individual control participant (at one time point) for the picture-scrambled contrast for the picture naming task. For picture naming, these regions include LSFG, bilateral MFG, bilateral IFG, LPCG, bilateral MTG, bilateral ITG, and bilateral fusiform gyrus. For semantic feature verification, Table 5 and Figure 3B show that regions that were consistently active across the eight participants included LSFG, LMFG, bilateral IFG, LPCG, bilateral MTG, LITG, bilateral AG, and bilateral fusiform regions. As the next step, we only included a region as a VOI in the connectivity analysis if each individual control subject showed significant activation (either at an uncorrected or FWE threshold).

**Table 4:** **Regions of activation across healthy control participants at one time point with** ***T*****-values for picture naming task**.

**Picture naming**	**Healthy controls**								
	**#4**	**#11**	**#13**	**#14**	**#67**	**#69**	**#70**	**#71**	**VOI**
**ANTERIOR LEFT**									
Left superior frontal gyrus	6.38	4.49	3.78	5.67	3.48	4.75	3.69	5.38	✓
Left middle frontal gyrus	5.65	4.70	6.25	5.57	4.34	3.56	5.14	7.72	✓
Left inferior frontal gyrus	8.05	6.16	5.80	9.36	4.44	4.84	8.68	7.66	✓
Left precentral gyrus	8.32	6.42	10.08	8.65	3.84	4.54	7.69	7.75	✓
**ANTERIOR RIGHT**									
Right superior frontal gyrus	5.98	4.14	5.93	3.23	3.40	3.32		5.34	
Right middle frontal gyrus	6.04	5.03	4.08	5.75	3.28	3.53	3.89	5.04	✓
Right inferior frontal gyrus	6.35	7.72	4.25	6.55	3.12	3.91	5.70	6.43	✓
Right precentral gyrus									
**POSTERIOR LEFT**									
Left superior temporal gyrus	3.80	3.77	8.33	4.26	4.54		3.18	4.69	
Left middle temporal gyrus	5.38	5.93	8.60	11.10	3.91	5.00	5.36	6.52	✓
Left inferior temporal gyrus	5.70	6.77	5.01	4.68	3.79	4.97	6.48	6.71	✓
Left fusiform gyrus	6.16	4.68	6.90	5.48	3.85	5.06	9.68	8.46	✓
Left supramarginal gyrus	6.76		8.41	3.23		3.73	4.35	3.39	
Left angular gyrus	4.64	6.03	4.17	4.69			3.29	6.42	
**POSTERIOR RIGHT**									
Right superior temporal gyrus	4.09	5.47	6.96	3.60		3.67		4.73	
Right heschls gyrus		5.62	3.72						
Right middle temporal gyrus	7.99	5.14	4.05	4.25	3.22	6.34	4.74	8.85	✓
Right inferior temporal gyrus	3.48	4.93	5.60	7.07	4.75	5.18	4.23	4.93	✓
Right fusiform gyrus	5.54	6.56	3.47	7.81	4.56	4.99	8.10	5.08	✓
Right supramarginal gyrus	3.65	4.03	7.24			3.88	4.02	4.13	
Right angular gyrus		3.78	5.15	3.39		3.46	3.35	4.89	

*Voxels active at p < 0.001 uncorrected have threshold at T values between 3.10 < T < 4.80 while voxels active at p < 0.05 family wise error correction (FWE) have threshold at T values T > 4.80. Red cells indicate significance at p < 0.05 FWE*.

**Table 5:** **Regions of activation across healthy control participants at one time point with** ***T*****-values for semantic feature verification task**.

**Semantic feature**	**Healthy controls**								
	**#4**	**#11**	**#13**	**#14**	**#67**	**#69**	**#70**	**#71**	**VOI**
**ANTERIOR LEFT**									
Left superior frontal gyrus	6.26	3.84	6.64	3.59	7.58	6.33	5.24	4.69	✓
Left middle frontal gyrus	4.45	4.44	5.36	4.24	4.91	8.18	7.59	4.26	✓
Left inferior frontal gyrus	6.86	7.67	7.72	5.94	9.26	11.16	6.94	7.27	✓
Left precentral gyrus	5.53	4.54	7.74	4.28	6.13	8.35	8.11	3.15	✓
**ANTERIOR RIGHT**									
Right superior frontal gyrus	3.12	3.17	4.40	3.37	4.84	3.28		3.21	
Right middle frontal gyrus	4.35	4.24	3.97		3.66	4.12	4.49	5.13	
Right inferior frontal gyrus	4.28	3.38	4.32	3.92	5.27	5.82	6.93	5.20	✓
Right precentral gyrus									
**POSTERIOR LEFT**									
Left superior temporal gyrus		4.06	4.63		4.68	9.19			
Left middle temporal gyrus	6.10	8.32	6.19	8.13	7.63	11.93	5.90	6.10	✓
Left inferior temporal gyrus	4.08	9.14	3.90	4.76	7.75	7.39	4.17	3.74	✓
Left fusiform gyrus	4.75	5.53	6.49	4.40	6.76	7.72	10.80	6.48	✓
Left supramarginal gyrus		4.67	4.15		5.00	6.13	4.57		
Left angular gyrus	3.35	6.32	3.61	4.09	5.30	6.15	6.13	5.51	✓
**POSTERIOR RIGHT**									
Right superior temporal gyrus		3.36	4.35			4.15	4.68		
Right Heschl's gyrus						3.78			
Right middle temporal gyrus	4.09	3.96	3.92	3.43	5.66	7.35	4.97	5.28	✓
Right inferior temporal gyrus		4.19	5.63	5.15	4.64	4.56	3.76	3.38	
Right fusiform gyrus	3.24	6.44	4.49	3.93	4.33	3.93	7.94	3.74	✓
Right supramarginal gyrus	3.21	3.53	4.11		4.54		4.78		
Right angular gyrus	3.67	3.32	3.70	3.40	5.07	4.58	4.32	4.52	✓

*Voxels active at p < 0.001 uncorrected have threshold at T values between 3.10 < T < 4.80 while voxels active at p < 0.05 family wise error correction (FWE) have threshold at T values T > 4.80. Red cells indicate significance at p < 0.05 FWE*.

#### Connectivity results for controls

**Figure 4:**
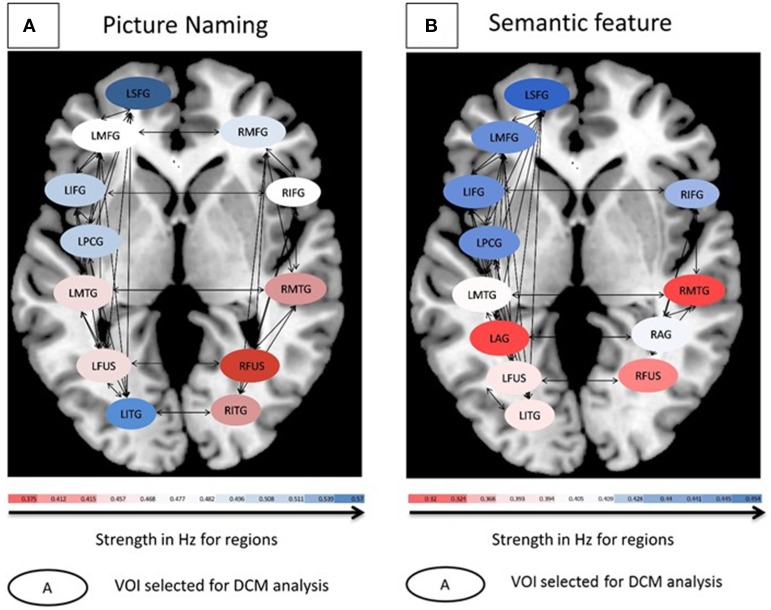
**(A)** Connectivity patterns for controls for the picture naming task. **(B)** Connectivity patterns for controls for the semantic feature verification task. For both tasks, average modulation for eight controls are shown. Oval shapes are the VOI's selected for model space, ranging from lowest (red) to highest (blue) modulation strengths. Intrinsic connections are shown in black arrows.

Each of the VOIs identified in Tables 4, 5 were then entered into BPA analysis as described above. Specifically, as our GLM consisted of three conditions (pictures, scrambled, and fixation), we used pictures for the driving (c-matrix) and modulatory (b-matrix) input as an effect of condition for our model space across both the tasks. Thus, the input used for DCM was the effect of condition “pictures.” The detailed specification of A, B, and C matrices are provided in Supplementary Table 2. For the picture naming task, there were 72 model combinations that were specified and for the semantic feature verification task, there were 76 model combinations that were specified. One-Way ANOVAs were performed on Ep.C (regions) for both the picture naming and semantic feature verification tasks separately. First, a One-Way ANOVA using Ep.C estimates as the dependent measure and input to regions as the independent measure across the eight participants for the picture naming task was found to be significant [*F*_(11, 564)_ = 4.00, *p* < 0.001]. Figure 4A shows that across the regions, LSFG, followed by LITG, LPCG, and LIFG had higher Ep.C values or modulations relative to RITG, RMTG and R fusiform regions (all *p* values significant at least <0.05).

Likewise, a one-way ANOVA using Ep.C estimates as the dependent measure and input to regions as the independent measure across the eight participants for the semantic feature verification task was found to be significant [*F*_(11, 596)_ = 11.3, *p* < 0.0001]. Figure 4B shows that across the regions, LSFG, followed by LMFG, LPCG, and LIFG had higher Ep.C values or modulations relative to R fusiform, RMTG and LAG regions (all *p* values significant at least <0.05). Due to the high number of different connections and limited number of participants, neither of the ANOVAs performed on the Ep.B (i.e., connections) values for the two tasks was significant. To summarize, the goal of the control analysis was to use the normal control VOIs and corresponding DCM analysis (which includes a broader set of VOIs than is available for patients) as a reference for the interpretation of patient DCM connectivity changes.

### Activation and connectivity results for patients after rehabilitation

#### fMRI activation results

**Figure 5:**
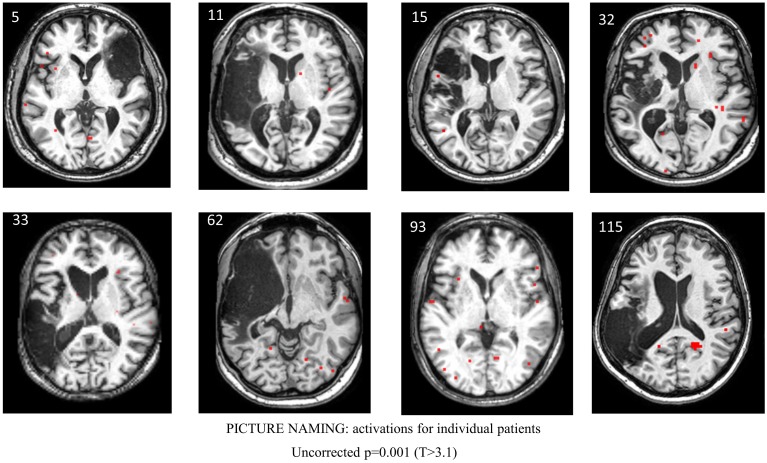
**Series of images showing individual patients' activation maps for the picture naming task illustrating the [post-rehabilitation (picture-scrambled)]–[pre-rehabilitation (picture-scrambled)] contrast for the trained category at** ***p*** **= 0.001 uncorrected**. Voxels above a threshold of *T* > 3.10 are shown.

Table 6 and Figure 5 show individual regions of activation that emerged for the post-rehabilitation > pre-rehabilitation contrast for the trained category on the picture naming task. Across patients, there were several regions that were consistently active in seven out of the eight patients as a function of rehabilitation that included LSFG, bilateral MFG, LPCG, and RMTG. Other regions that were active in most (>6) patients included RSFG, bilateral IFG, bilateral SMG, LAG, and RSTG. When compared to Table 4, it is apparent that several of these regions (LSFG, bilateral MFG, LPCG, bilateral IFG, and RMTG) are active in all normal controls, indicating that as a function of treatment, several normally-engaged language regions are further recruited after rehabilitation.

**Table 6:** **Regions of activation across patients in the post-rehabilitation > pre-rehabilitation contrast with** ***T*****-values for the picture naming task**.

**Picture naming**	**Patients: trained category**								
	**Post > Pre**								**Count**
	**#05**	**#11**	**#15**	**#32**	**#33**	**#62**	**#93**	**#115**	
**ANTERIOR LEFT**									
Left superior frontal gyrus	4.24		4.65	3.55	4.16	3.41	3.73	3.56	7
Left middle frontal gyrus	5.84		3.73	4.37	5.86	3.47	3.75	3.80	7
Left inferior frontal gyrus	4.26		5.70	3.32	4.78	3.69	4.25		6
Left precentral gyrus	4.72	3.18	3.77	3.75	3.77	3.63	4.24	3.54	8
**ANTERIOR RIGHT**									
Right superior frontal gyrus	3.78		4.88	3.30	5.74		4.28	3.96	6
Right middle frontal gyrus	3.62		4.57	3.51	4.80	3.36	4.96	3.79	7
Right inferior frontal gyrus	3.40		4.81	4.87	6.25		3.66	3.60	6
Right precentral gyrus									0
**POSTERIOR LEFT**									
Left superior temporal gyrus	4.08		6.98		3.84	3.24	4.06		5
Left middle temporal gyrus	6.11		8.44	3.19	3.94	3.39	4.07		6
Left inferior temporal gyrus			4.59	3.45			4.78		3
Left fusiform gyrus		3.16	6.60	3.46			4.64		4
Left supramarginal gyrus	3.18		4.81	3.36	3.84	3.72	3.83		6
Left angular gyrus	3.93		4.79	3.95	3.99	3.51	3.92		6
**POSTERIOR RIGHT**									
Right superior temporal gyrus			4.25	3.53	4.71	3.37	3.72	3.54	6
Right Heschl's gyrus							3.12		1
Right middle temporal gyrus	4.20		4.61	4.77	4.14	3.33	3.67	3.31	7
Right inferior temporal gyrus			3.91	3.99	3.15	3.17	3.58		5
Right fusiform gyrus			3.27	3.99			3.56		3
Right supramarginal gyrus	3.98		3.40		4.28	3.50	4.51	3.86	6
Right angular gyrus	3.60		3.54		3.57		3.75	3.45	5

*Voxels active at p < 0.001 uncorrected have threshold at T values between 3.10 < T < 4.80 while voxels active at p < 0.05 FWE have threshold at T values T > 4.80. Red cells indicate significance at p < 0.05 FWE. The last column indicates the number of patients that showed activation that exceeded the uncorrected threshold*.

**Figure 6:**
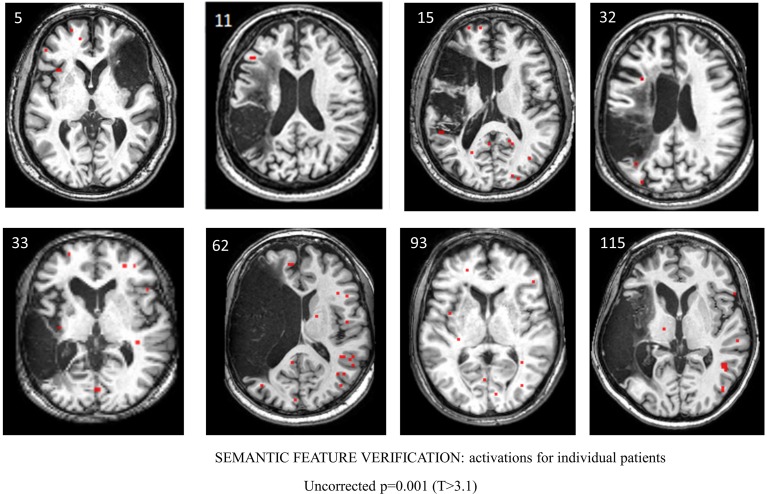
**Series of images showing individual patients' activation maps for the semantic feature verification task illustrating the [post-rehabilitation (picture-scrambled)]–[pre-rehabilitation (picture-scrambled)] contrast for the trained category at** ***p*** **= 0.001 uncorrected**. Voxels above a threshold of *T* > 3.10 are shown.

For the semantic feature verification task, Table 7 and Figure 6 show individual regions of activation that emerged for the post-rehabilitation > pre-rehabilitation contrast for the trained category. While changes in patient activation were less consistent in this task, seven out of eight patients showed changes as a function of rehabilitation in RMFG, RMTG, and RAG. Other regions that were active in at least six patients included LPCG, RSFG, RIFG, LMTG, and RSTG. When compared to Table 5, some of these regions were also active in normal controls including LPCG, RIFG, RMTG, and LMTG. Interestingly, there were several regions in the right hemisphere (RMFG, RSFG, RSTG) that were not consistently active in the normal controls but emerged in the post > pre-rehabilitation contrast.

**Table 7:** **Regions of activation across patients in the post-rehabilitation > pre-rehabilitation contrast with** ***T*****-values for the semantic feature verification task**.

**Semantic feature**	**Patients: trained category**								
	**Post > Pre**								**Count**
	**#05**	**#11**	**#15**	**#32**	**#33**	**#62**	**#93**	**#115**	
**ANTERIOR LEFT**									
Left superior frontal gyrus	4.00		5.00	3.18	3.33			4.19	5
Left middle frontal gyrus	5.58		3.37		3.11			3.24	4
Left inferior frontal gyrus		3.73		3.72	3.81		3.77		4
Left precentral gyrus	3.27	3.92	5.33	3.36	3.90			5.08	6
**ANTERIOR RIGHT**									
Right superior frontal gyrus	5.89	3.86	5.76	3.47	3.83			4.56	6
Right middle frontal gyrus	3.85		4.93	3.70	5.10	4.54	3.19	4.67	7
Right inferior frontal gyrus	4.87		3.34	3.42	4.65		4.61	4.68	6
Right precentral gyrus									0
**POSTERIOR LEFT**									
Left superior temporal gyrus	3.45	3.18	4.22	3.58	4.12				5
Left middle temporal gyrus	3.60	3.24	4.66	3.57	4.21		3.42		6
Left inferior temporal gyrus			3.17		3.86			3.17	3
Left fusiform gyrus				3.61	3.74			3.84	3
Left supramarginal gyrus	3.73	4.09	4.49				3.23		4
Left angular gyrus	4.64		3.50	3.86	3.11		3.18		5
**POSTERIOR RIGHT**									
Right superior temporal gyrus	3.12	3.57		3.30	3.79		4.30	5.07	6
Right Heschl's									0
Right middle temporal gyrus	3.57	3.12	4.93		3.70	5.04	3.95	3.25	7
Right inferior temporal gyrus			5.32		3.29		3.73	3.11	4
Right fusiform gyrus			3.76			6.29	3.34	4.22	4
Right supramarginal gyrus			4.04	4.04	3.45		4.01	3.89	5
Right angular gyrus	3.68		5.09	3.22	3.69	4.70	3.46	4.36	7

*Voxels active at p < 0.001 uncorrected have threshold at T values between 3.10 < T < 4.80 while voxels active at p < 0.05 FWE have threshold at T values T > 4.80. Red cells indicate significance at p < 0.05 FWE. The last column indicates the number of patients that showed activation that exceeded the uncorrected threshold*.

It should be noted that all of these regions identified above are those that emerged as regions that were more active after rehabilitation relative to before rehabilitation. This analysis does not reflect regions that were active before and after rehabilitation and that may have changed as a function of rehabilitation but did not cross the activation thresholds. The question of how regions that may have been active before and after rehabilitation and may have been modulated is addressed in the connectivity analysis. Additionally, a complete explanation of activation for the untrained categories is beyond the scope of this paper and is provided in Supplementary Tables 3, 4.

#### Connectivity results for patients

Recall that for each patient, a common active VOI present at both the pre- and post-rehabilitation scan (at *p* < 0.001 uncorrected) was extracted for each of the two tasks using the same procedure as that described for normal controls. These VOIs largely overlapped with the VOIs extracted for the normal controls, although, across patients, the specific set of VOIs were different. Patient #62 was excluded from DCM analysis as there were no common active VOIs present in both scans. See Table 8 for VOIs selected for patients and supplementary Table 5 for a complete description of the full model space for each patient across the two tasks.

**Table 8:** **Regions of interest for patients (active at both pre and post-rehabilitation) selected for DCM analysis for the picture naming task and semantic feature task**.

**PICTURE NAMING**						
#05	#11	#15	#32	#33	#93	#115
LAG	LIFG	LAG	LFUSI	LIFG	LIFG	LMFG
LIFG	LITG	LIFG	LIFG	LMFG	LITG	LFUSI
LPCG	LPCG	LSFG	LITG	RAG	LPCG	LIFG
	RIFG	RIFG	RFUSI	RIFG	LSFG	LPCG
	RMFG	RMFG	RIFG	RMFG	RAG	RFUSI
			RITG		RITG	RIFG
					RSTG	RMFG
						RSTG
**SEMANTIC FEATURE**						
#05	#11	#15	#32	#33	#93	#115
LIFG	LIFG	LIFG	LIFG	LFUSI	LSFG	LMFG
RIFG	LMFG	RIFG	LMFG	LIFG	LIFG	LIFG
RMFG	LMTG		LMTG	LMFG	LMTG	LITG
RSFG	LPCG		RIFG	LSFG	RIFG	LPCG
	LSFG		RITG	RAG	RMTG	RAG
	RAG		RMFG	RFUSI	RAG	RIFG
	RIFG		RMTG	RMTG		RMFG
	RMFG		RSFG			RMTG

*The regions are thresholded at p < 0.001 uncorrected*.

To examine rehabilitation-induced modulations in patients, separate, two-factor (trained and untrained categories) repeated measures ANOVAs using Ep.B (estimates on connections) and Ep.C (estimates on regions) as dependent measures with rehabilitation outcome (effect size for trained and untrained assessed category) as the covariate were performed for each task (picture naming and semantic feature). Specific regions and connections were collapsed across patients in these analyses.

For the picture naming task, when estimates on connections (Ep.B) were examined, the effect of rehabilitation was not significant [*F*_(1, 177)_ = 1.6; *p* = ns], the interaction between rehabilitation and the effect size covariate was also not significant [*F*_(1, 177)_ = 0.82; *p* = ns] and finally, the interaction between rehabilitation and category also was not significant [*F*_(1, 177)_ = 0.59; *p* = ns]. Second, when estimates on regions (Ep. C) were examined, the effect of rehabilitation was significant [*F*_(1, 59)_ = 9.9; *p* < 0.01]; while the interaction between rehabilitation and effect size covariate was not significant [*F*_(1, 59)_ = 3.1; *p* = 0.08], the interaction between rehabilitation and category was significant [*F*_(1, 59)_ = 4.6; *p* < 0.05]. *Post-hoc* LSD tests showed lower Ep.C values after rehabilitation for the trained category but not for the untrained category (all differences significant at *p* < 0.05).

Similar analyses were performed for the semantic feature task. First, when estimates on connections (Ep.B) were examined, the effect of rehabilitation modulation was significant [*F*_(1, 193)_ = 18.4; *p* < 0.0001], the interaction between rehabilitation and effect size covariate also was significant [*F*_(1, 193)_ = 108.7; *p* < 0.0001], and finally, the interaction between rehabilitation and category was significant [*F*_(1, 193)_ = 8.15; *p* < 0.01]. *Post-hoc* LSD tests showed higher Ep.B values after rehabilitation for the untrained category than the trained category (all differences significant at *p* < 0.05). When examining the estimates on regions (Ep.C), the effect of rehabilitation was not significant [*F*_(1, 59)_ = 0.91; *p* = ns], and the interaction between rehabilitation and the effect size covariate was not significant [*F*_(1, 59)_ = 0.42; *p* = *ns*]. Finally, the interaction between rehabilitation and category also was not significant [*F*_(1, 59)_ = 0.10; *p* = ns]. These results, when significant, point to lower BPA values for the trained relative to the untrained category. They, however, do not explain which regions change their connectivity as a function of rehabilitation and if the network changes are consistent from patient to patient.

One likely reason for the lack of significance in some of the analyses is inter-participant variability. Therefore, individual repeated measures ANOVAs on pre- and post-estimates as dependent measures for both Ep.B and Ep.C and participants as the independent variable were performed for the trained category for the picture naming and semantic feature tasks (see Supplementary Section). All the analyses showed a significant patient-by-rehabilitation interaction, indicating that certain patients showed greater differences between their pre- and post-treatment Ep.B and Ep.C estimate values for both tasks. Therefore, it is likely that inter-subject differences overshadowed any group level differences.

**Figure 7:**
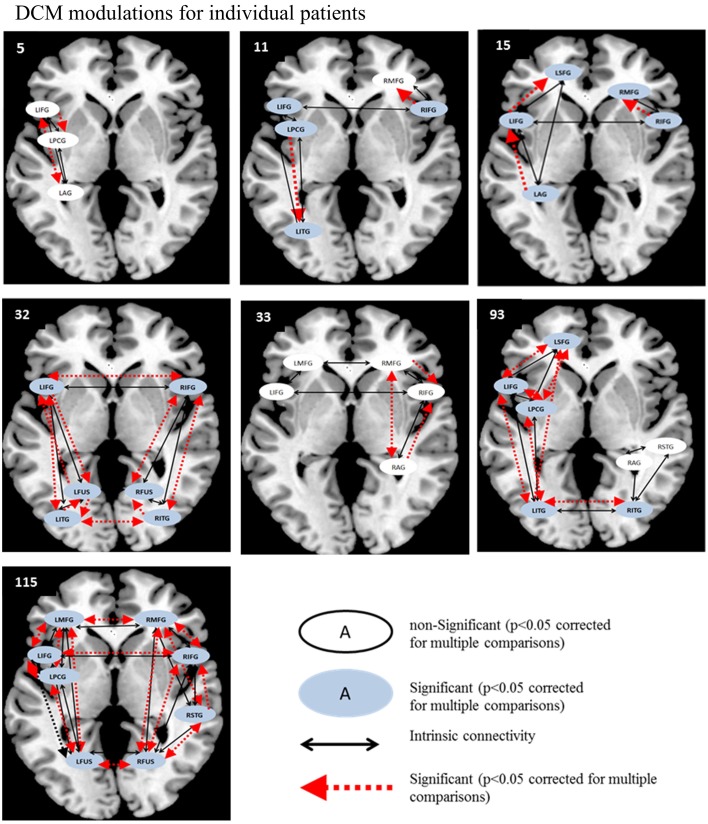
**Series of images show individual patients' connectivity networks as a function of rehabilitation for the trained category on the picture naming task**. Regions with significant modulations are shown in blue ovals while regions with non-significant modulations are shown in white ovals. Intrinsic connections are shown in black arrows while connections with significant modulations are shown in red arrows.

Therefore, as in the fMRI analysis, individual patient data were analyzed before and after rehabilitation as paired *t*-tests for individual participants. For each patient, paired *t*-tests were performed on pre-rehabilitation and post-rehabilitation averaged BPA Ep.B (and Ep.C values) over the entire model space with a significance criterion set at *p* < 0.05. As displayed in Figure 7, for the picture naming task, LIFG was the most consistently active VOI in the pre- and post-rehabilitation scans and the most consistently significantly modulated region as a function of rehabilitation (5/7 patients). Next, two other regions that were consistently active across participants at the pre- and post-rehabilitation scans and were consistently significantly modulated as a function of rehabilitation were LPCG (3/4 patients) and RIFG (4/5 patients). Notably, even though few patients showed consistent activation in LITG, RITG, and LSFG in the pre- and post-rehabilitation scans, these regions were modulated as a function of rehabilitation when they were active. In terms of connections, RIFG-RMFG (4/4 patients) was the most consistently significantly modulated connection as a function of rehabilitation. In addition, LIFG-LPCG (3/4 patients) and LIFG-LITG (3/3 patients) also showed a significant modulation as a function of rehabilitation. Importantly, LITG, LSFG, LIFG, and RIFG were also all regions that were modulated for normal controls.

**Figure 8:**
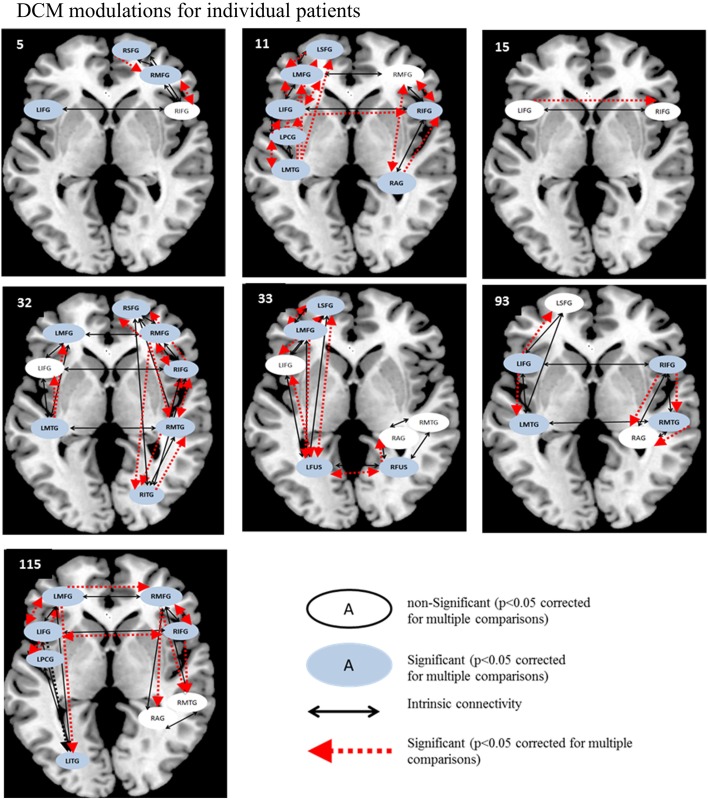
**Series of images showing individual patients' connectivity networks as a function of rehabilitation for the trained category on the semantic feature verification task**. Regions with significant modulations are shown in blue ovals while regions with non-significant modulations are shown in white ovals. Intrinsic connections are shown in black arrows while connections with significant modulations are shown in red arrows.

Likewise, for the semantic feature verification task (see Figure 8), LIFG was the most consistently active region and significantly modulated in 4/7 patients. Interestingly, RIFG was consistently active and significantly modulated in 4/6 patients and LMFG was consistently active and significantly modulated in 4/4 patients. Similarly, LMTG was an active VOI in only three patients but was significantly modulated in all three of them. In terms of connections, RIFG-RMFG was significantly modulated in all four patients who had these regions in their network, as was LIFG-LMFG (4/4 patients), LIFG-RIFG (3/3 patients), and RIFG-RMTG (3/3) patients. Most of the regions that were modulated as a function of rehabilitation in the patients were similar to those in the healthy control network for this task, including LSFG, LMFG, LIFG, LPCG, and RIFG.

## Discussion

The goal of this study was to examine changes in BOLD signal activation and in effective connectivity as a function of neurorehabilitation across eight patients with aphasia. All patients presented with naming impairments, received rehabilitation to improve their naming skills and showed changes in activation and connectivity as a function of the intervention. Two tasks, picture naming and semantic feature verification, which closely aligned with the rehabilitation approach, were used for the BOLD signal change and connectivity analysis and to examine brain-behavior relationships before and after rehabilitation. The main observations of the study were as follows: (a) all patients improved as a function of rehabilitation, and improvements across patients were greater for the trained categories than the untrained categories, (b) in terms of the fMRI changes in activation, there were several regions such as LIFG, RIFG, LPCG, LMFG, RMFG, LMTG, and RMTG that were consistently active in normal controls and in several of post > pre-rehabilitation comparisons for patients, and (c) in terms of connectivity changes across patients, LIFG was the most modulated region, independent of task and as a function of rehabilitation. Each of these results will be addressed in greater depth below.

As noted in the introduction, naming deficits are pervasive in individuals with aphasia and a semantic feature-based treatment that emphasizes semantic feature analysis and phonological access was effective in improving word retrieval even in individuals with chronic aphasia. While we have examined generalization to untrained items in our previous work, in this study we instead chose to examine the changes on the trained category (including both trained and untrained items) only using two tasks (picture naming and semantic feature verification) that were germane to the rehabilitation. Results showed that a semantic-feature based naming intervention resulted in improvements in naming function in all patients on the trained category. While the efficacy of this intervention has been examined before (Kiran and Thompson, 2003; Kiran, 2007, 2008; Kiran and Johnson, 2008), the present results further extend the validity of this rehabilitation approach to facilitate language recovery in patients with aphasia. It should be noted that irrespective of which category was trained, behavioral improvements in the trained category was higher relative to untrained monitored categories.

Next, changes in patterns of BOLD signal activation as a function of rehabilitation revealed that regions such as LIFG, RIFG, LPCG, LMFG, RMFG, LMTG, RMTG, LAG, and RAG showed greater activation after rehabilitation for picture naming. For semantic feature verification, a subset of the above regions were active including RIFG, LPCG, RMFG, LMTG, RMTG, as well as RSTG. Several of these regions were also significantly activated in the healthy controls indicating that these regions comprise a set of core regions that are required for normal language processing and to subserve rehabilitation-induced language recovery. While the role of LIFG and LMTG is well understood for semantic processing and word retrieval (Seghier et al., 2004; Vigneau et al., 2006; Binder et al., 2009; van Oers et al., 2010; Cappa, 2012; Visser et al., 2012; Jefferies, 2013), LMFG has been implicated in both semantic processing (Binder et al., 2009) as well as in domain general processing of tasks with increased difficulty (Fedorenko et al., 2013). Also, LSFG has been implicated in semantic processing (Binder et al., 2009). Likewise, LPCG has been observed in previous studies to emerge as a region with increased activation as a function of a semantic-based naming treatment (Marcotte et al., 2012).

Interestingly, the role of the RIFG is less well documented for normal healthy controls (Wierenga et al., 2008). In a recent metaanalytic review, Vigneau et al. (2011) noted that few studies reported unilateral right hemisphere activation during lexical-semantic processing; rather, most activation of right frontal regions tended to be bilateral activation. RIFG and RMTG, however, have been reported fairly extensively in patients with left hemisphere injury (Voets et al., 2006; Crosson et al., 2007; Harnish et al., 2008; van Oers et al., 2010). In another study, we have demonstrated that RMFG is part of a network involved in recovered semantic processing in patients with aphasia (Sims et al., under review). In line with this, left fronto-parietal cortex and right middle frontal cortex (and medial frontal cortex) may be critical regions involved in word/sentence comprehension in patients with aphasia as shown in a recent study examining intrinsic functional connectivity (Zhu et al., 2014).

There were also regions such as bilateral ITG and fusiform gyrus that were active for controls but not consistently observed in the post > pre-rehabilitation comparisons across patients. These regions have been implicated in perceptual processing of visual objects (Soldan et al., 2010) including their featural attributes (Zannino et al., 2010; Tyler et al., 2013) and in processing of semantic information for words and pictures (Seghier and Price, 2011) in normal individuals. Since these regions were not consistently active across patients in the post > pre-rehabilitation contrasts, these regions may not yet be integrated into the normal picture naming or semantic feature verification language network for individuals who do not show these regions engaged as a function of rehabilitation.

Next, we examined changes in connectivity across patients as a function of rehabilitation. Because patients' individual responsiveness to rehabilitation was varied, as was their lesion site and size, individual model spaces were created for each patient based on regions that were active before and after rehabilitation. First, group level analyses revealed the effects of rehabilitation on modulation with lower modulation for regions and connections for the trained compared to the untrained category. Unfortunately, these analyses were not significant in all the comparisons, and further, did not indicate which regions/connections were subject to greater modulation as a function of rehabilitation. Therefore, individual patient network analyses proved to be more useful. Across patients, regions including LIFG, RIFG, and LMFG were active and modulated for both tasks; LSFG, LITG, and LFUS were active and modulated for picture naming; and RMFG, LMTG, and RMTG were active and modulated for semantic feature verification as a function of rehabilitation. Notably, specific modulations of these regions within individual patient networks varied as a function of lesion size and site. Nonetheless, LIFG was the most consistently modulated region, independent of task and as a function of rehabilitation, followed by RIFG and LMFG. These changes in modulation after rehabilitation are consistent with the changes in patterns of activation for individual patients, indicating that regions identified with increased activation after rehabilitation are also correspondingly significantly modulated within the network. These preliminary results indicate the presence of nodes of change within the language network across patients.

One methodological note worth pointing out here is that “new” regions that emerge as a function of rehabilitation would be identified in the post > pre-rehabilitation contrast, but may have been missed in the DCM analysis since only regions that were active at the post > pre-rehabilitation contrasts were considered. As an example, Table 7 shows greater activation in RSFG for 6/8 patients for the semantic feature verification task; however, as seen in Figure 8, only two patients show this region as significantly active in the post > pre-rehabilitation contrast and significantly modulated in the DCM analysis. A complete discussion of which “new” regions emerge as a function of rehabilitation compared to which existing regions alter their modulation due to rehabilitation is out of the scope of this paper. Nonetheless, the present study provides preliminary evidence that such a distinction may be captured in carefully constructed fMRI and connectivity experiments.

The results of our study are different from several recent studies described in the introduction that have not demonstrated left hemisphere substrates subsequent to rehabilitation. Instead, the present results are consistent with studies that suggest that traditional language regions such as IFG, MTG, and PCG are also engaged subsequent to improvements in behavior as a function of rehabilitation.

It should be noted that individual patient networks as a function of rehabilitation (at two time points) look qualitatively different from the normal language network (captured at one time point). These results can be interpreted as follows. First, task-specific modulation for normal controls indicated a bilateral network for picture naming and a left-lateralized network for semantic feature verification. These results are consistent with findings of an fMRI study that examined semantic categorization (predominantly semantic processing) and rhyme detection (predominantly phonological processing) in normal individuals (Seghier et al., 2004). Like the present results, Seghier et al. found that while both tasks revealed left frontal activation, phonological processing (closer to our picture naming task) revealed more bilateral activation than semantic processing which was predominantly left lateralized. In our study, while not all patients show differential lateralization in their networks as a function of rehabilitation, some patients show patterns similar to the control network.

Second, the normal network can be interpreted as a network engaged in successful language processing (either picture naming or semantic processing) and the corresponding modulations of regions indicative of their relative contributions within the network. The individual patient networks, in contrast, are indicative of reorganized or altered networks that have not completely returned to their normal-like function (Teki et al., 2013). Further, the precise implications of the modulation of LIFG in this study are difficult to ascertain as there are too few participants and the patterns across these individuals are variable. Nonetheless, there are a few interesting observations that warrant future examination. First, patients who showed some spared LIFG also showed significant changes in activation as a function of rehabilitation as well as significant modulation (Patients # 32, #15, #93; however see Patient #33) suggesting that the presence of modulation in the LIFG depends on the degree of spared LIFG. Second, patients with large lesions (Patients # 32, #11, and #115) also showed significant modulation of the RIFG, suggesting that for such patients, RIFG is an important part of the reorganized network, a finding that is consistent with previous research (Turkeltaub et al., 2011). While the results of this study are preliminary, they set the stage for future examinations of the brain-behavior relationships as a function of neurorehabilitation and allow perusal of factors that influence the impairment and corresponding recovery.

An important limitation of this study is that changes in connectivity are not interpreted in terms of magnitude and directionality as there are too few patients to draw any meaningful conclusions. Future studies with larger groups of homogenous patients that can systematically examine and interpret magnitude and directionality of change need to be undertaken.

There are several theoretical and clinical implications of these results. From a theoretical standpoint, even with the inherent variability across patients, the results underscore the importance of LIFG in the retrained language network in post-stroke patients with aphasia. This finding is not surprising, and has been a consistent observation in most studies examining the nature of language recovery in post-stroke aphasia (Fridriksson, 2010; Marcotte and Ansaldo, 2010; Rochon et al., 2010; Fridriksson et al., 2012; Sebastian et al., 2012; Sims et al., under review). All of these studies are fMRI activation studies, and the results of the present study demonstrate that when using effective connectivity analysis across individual patients, when undamaged, LIFG is an important node of a retrained language network. Another important observation in the present study is the role of the RIFG and connections between the RIFG and RMFG that were consistently modulated in patients. While the discussion of the role of right hemisphere homologs has been debated in the literature (Winhuisen et al., 2007; Raboyeau et al., 2008; van Oers et al., 2010) with suggestions that it may play a more complementary or supportive role (Turkeltaub et al., 2011), the present results indicate that these regions are also an important part of the retrained language network and are associated with improved language function at least for some individuals.

From a clinical perspective, the results highlight the importance of language rehabilitation shaping neuroplasticity even in chronic stroke patients with residual aphasia. Despite obvious differences in patterns of activation that are constrained by individual patient lesions, this study provides tentative support for the assumption that rehabilitation promotes a damaged brain to reorganize to support language processing abilities. The remarkable consistency across patients in terms of the modulation within the networks (LIFG, LPCG, and LMTG) indicates that there is a systematic way the network reorganizes for language recovery as a function of rehabilitation.

The small number of healthy controls and patients involved in the study limits the conclusions that can be drawn from the study. Also, the inherent variability in lesion and behavioral profiles across the participants warranted all the behavioral, neuroimaging and connectivity data to be analyzed at the individual participant level. While this type of analysis (i.e., case-series approach), required several modifications to the traditional group level analysis, the explicit attempt to account for and detail the inherent variability across the patients with aphasia is a clear strength of this study. Within the patient group, there are several levels of controls and replications that have been incorporated in the experimental design which allow meaningful interpretations to be drawn from the study (Kiran et al., 2013). Future studies in larger samples can examine how these regions change in comparison to the amount of rehabilitation outcome. Finally, the neurorehabilitation approach described in this study allowed the standardization of the methods across patients even though the rehabilitation was targeted at the individual patient's impairment. Ultimately, these results are important for understanding the brain-behavior relationship during and after the process of rehabilitation of language processing after a stroke.

### Conflict of interest statement

The authors declare that the research was conducted in the absence of any commercial or financial relationships that could be construed as a potential conflict of interest.

## Footnotes

^1^#5 presented with crossed-aphasia, although it is unclear whether he was left or right hemisphere dominant for language prior to his stroke. Given the inherent variability across all participants, we chose to include this patient's data in the study as all data were analyzed individually, and the observation of similar results in a patient with a right hemisphere stroke and aphasia strengthens this study.

^2^Effect sizes and percent change reported here are the average of the trained atypical example and untrained typical examples. A full examination of the extent of generalization to typical examples is out of the scope of this study.
