# Supplementary material for: Changes in task-based effective connectivity in language networks following rehabilitation in post-stroke patients with aphasia
Source: Front Hum Neurosci. 2015 Jun 9;9:316. doi: 10.3389/fnhum.2015.00316 (PMC4460429; doi:10.3389/fnhum.2015.00316)
Supplement: Supplementary file 1 [file DataSheet1.DOCX]

1. **Appendix**

**Table of abbreviations**

| DCM | Dynamic Causal Modeling |
| --- | --- |
| BOLD signal | Blood Oxygen Level Dependent Signal |
| LIFG | Left Inferior Frontal Gyrus |
| RIFG | Right Inferior Frontal Gyrus |
| SFG | Superior Frontal Gyrus |
| MFG | Middle Frontal Gyrus |
| PCG | Precentral Gyrus |
| MTG | Middle Temporal Gyrus |
| STG | Superior Temporal Gyrus |
| IPL | Inferior Parietal Lobule |
| ITG | Inferior Temporal Gyrus |
| SMG | Supramarginal Gyrus |
| AG | Angular Gyrus |
| WAB-AQ | Western Aphasia Battery-AQ |
| CLQT | Cognitive Linguistic Quick Test |
| PAPT | Pyramids And Palm Trees |
| BNT | Boston Naming Test |
| BPA | Bayesian Parameter Average |
