# Supplementary material for: Changes in task-based effective connectivity in language networks following rehabilitation in post-stroke patients with aphasia
Source: Front Hum Neurosci. 2015 Jun 9;9:316. doi: 10.3389/fnhum.2015.00316 (PMC4460429; doi:10.3389/fnhum.2015.00316)
Supplement: Supplementary file 2 [file DataSheet2.DOCX]

**Changes in task based effective connectivity in language networks following rehabilitation in post-stroke patients with aphasia**

**Swathi Kiran^1^, Kushal Kapse^1^, Peter Glynn^2^**

^1^Aphasia Research Laboratory, Sargent College, Speech Language and Hearing Sciences, Boston University, Boston MA, USA

^2^Northwestern University, Feinberg School of Medicine, Chicago IL, USA

*** Correspondence:** Swathi Kiran, Aphasia Research Laboratory, Sargent College, Speech Language and Hearing Sciences, Boston University, 635 Commonwealth avenue, Rm 326 Boston MA 02215, USA. [kirans@bu.edu](mailto:kirans@bu.edu)

1. **Supplementary Material**

**Table 1: Information about eight normal controls that were recruited from two studies. Study 1 included 4 healthy controls (Age: 46-57 years, 2 females) and Study 2 included 4 healthy controls (Age: 46 – 69 years, 2 females).**

| Picture Naming | Study 1 (n = 4) | Study 2 (n = 4) |
| --- | --- | --- |
| #volumes | 113.00 | 196.00 |
| time/run | 4mins42sec | 8mins24sec |
| TR (sec) | 2.50 | 2.57 |
| runs | 4.00 | 2.00 |
| picture duration (sec) | 5.00 | 4.00 |
| scrambled duration (sec) | 3.00 | 4.00 |
| fixation duration (sec) | 2,4 | 2,4 |
| #pictures | 20.00 | 54.00 |
| #scrambled | 20.00 | 18.00 |
| #fixation | 20,20 | 36,36 |
| design | randomized event related with jittered ISI | randomized event related with jittered ISI |
|  | | |
| Semantic Feature Verification | Study 1 (n = 4) | Study 2 (n = 4) |
| #volumes | 130.00 | 224.00 |
| time/run | 5min24sec | 9mins36sec |
| TR (sec) | 2.50 | 2.57 |
| runs | 4.00 | 2.00 |
| picture duration (sec) | 5.00 | 5.00 |
| scrambled duration (sec) | 5.00 | 5.00 |
| fixation duration (sec) | 2,4 | 2,4 |
| #pictures | 20.00 | 54.00 |
| #scrambled | 20.00 | 18.00 |
| #fixation | 20,20 | 36,36 |
| design | randomized event related with jittered ISI | randomized event related with jittered ISI |

**Table 2. The full DCM model space for each individual control for both the picture naming task and the semantic feature verification task. Note: B-matrix= input to connection=modulation, C-matrix=input to region=perturbation; A-matrix= model specification will contain within hemisphere regions connected to each other, while only homologous regions connected between hemisphere.**

| **Picture Naming** | | | **Semantic Feature** | | |
| --- | --- | --- | --- | --- | --- |
| **model#** | **B-matrix** | **C-matrix** | **model#** | **B-matrix** | **C-matrix** |
| **1** | **LFUSI-LIFG** | **LFUSI** | **1** | **LAG-LFUSI** | **LAG** |
| **2** | **LFUSI-LITG** | **LFUSI** | **2** | **LAG-LIFG** | **LAG** |
| **3** | **LFUSI-LMFG** | **LFUSI** | **3** | **LAG-LITG** | **LAG** |
| **4** | **LFUSI-LMTG** | **LFUSI** | **4** | **LAG-LMFG** | **LAG** |
| **5** | **LFUSI-LPCG** | **LFUSI** | **5** | **LAG-LMTG** | **LAG** |
| **6** | **LFUSI-LSFG** | **LFUSI** | **6** | **LAG-LPCG** | **LAG** |
| **7** | **LIFG-LFUSI** | **LIFG** | **7** | **LAG-LSFG** | **LAG** |
| **8** | **LIFG-LITG** | **LIFG** | **8** | **LFUSI-LAG** | **LFUSI** |
| **9** | **LIFG-LMFG** | **LIFG** | **9** | **LFUSI-LIFG** | **LFUSI** |
| **10** | **LIFG-LMTG** | **LIFG** | **10** | **LFUSI-LITG** | **LFUSI** |
| **11** | **LIFG-LPCG** | **LIFG** | **11** | **LFUSI-LMFG** | **LFUSI** |
| **12** | **LIFG-LPCG** | **LIFG** | **12** | **LFUSI-LMTG** | **LFUSI** |
| **13** | **LITG-LFUSI** | **LITG** | **13** | **LFUSI-LPCG** | **LFUSI** |
| **14** | **LITG-LIFG** | **LITG** | **14** | **LFUSI-LSFG** | **LFUSI** |
| **15** | **LITG-LMFG** | **LITG** | **15** | **LIFG-LAG** | **LIFG** |
| **16** | **LITG-LMTG** | **LITG** | **16** | **LIFG-LFUSI** | **LIFG** |
| **17** | **LITG-LPCG** | **LITG** | **17** | **LIFG-LITG** | **LIFG** |
| **18** | **LITG-LSFG** | **LITG** | **18** | **LIFG-LMFG** | **LIFG** |
| **19** | **LMFG-LFUSI** | **LMFG** | **19** | **LIFG-LMTG** | **LIFG** |
| **20** | **LMFG-LIFG** | **LMFG** | **20** | **LIFG-LPCG** | **LIFG** |
| **21** | **LMFG-LITG** | **LMFG** | **21** | **LIFG-LSFG** | **LIFG** |
| **22** | **LMFG-LMTG** | **LMFG** | **22** | **LITG-LAG** | **LITG** |
| **23** | **LMFG-LPCG** | **LMFG** | **23** | **LITG-LFUSI** | **LITG** |
| **24** | **LMFG-LSFG** | **LMFG** | **24** | **LITG-LIFG** | **LITG** |
| **25** | **LMTG-LFUSI** | **LMTG** | **25** | **LITG-LMFG** | **LITG** |
| **26** | **LMTG-LIFG** | **LMTG** | **26** | **LITG-LMTG** | **LITG** |
| **27** | **LMTG-LITG** | **LMTG** | **27** | **LITG-LPCG** | **LITG** |
| **28** | **LMTG-LMFG** | **LMTG** | **28** | **LITG-LSFG** | **LITG** |
| **29** | **LMTG-LPCG** | **LMTG** | **29** | **LMFG-LAG** | **LMFG** |
| **30** | **LMTG-LSFG** | **LMTG** | **30** | **LMFG-LFUSI** | **LMFG** |
| **31** | **LPCG-LFUSI** | **LPCG** | **31** | **LMFG-LIFG** | **LMFG** |
| **32** | **LPCG-LIFG** | **LPCG** | **32** | **LMFG-LITG** | **LMFG** |
| **33** | **LPCG-LITG** | **LPCG** | **33** | **LMFG-LMTG** | **LMFG** |
| **34** | **LPCG-LMFG** | **LPCG** | **34** | **LMFG-LPCG** | **LMFG** |
| **35** | **LPCG-LMTG** | **LPCG** | **35** | **LMFG-LSFG** | **LMFG** |
| **36** | **LPCG-LSFG** | **LPCG** | **36** | **LMTG-LAG** | **LMTG** |
| **37** | **LSFG-LFUSI** | **LSFG** | **37** | **LMTG-LFUSI** | **LMTG** |
| **38** | **LSFG-LIFG** | **LSFG** | **38** | **LMTG-LIFG** | **LMTG** |
| **39** | **LSFG-LITG** | **LSFG** | **39** | **LMTG-LITG** | **LMTG** |
| **40** | **LSFG-LMFG** | **LSFG** | **40** | **LMTG-LMFG** | **LMTG** |
| **41** | **LSFG-LMTG** | **LSFG** | **41** | **LMTG-LPCG** | **LMTG** |
| **42** | **LSFG-LPCG** | **LSFG** | **42** | **LMTG-LSFG** | **LMTG** |
| **43** | **RFUSI-RIFG** | **RFUSI** | **43** | **LPCG-LAG** | **LPCG** |
| **44** | **RFUSI-RITG** | **RFUSI** | **44** | **LPCG-LFUSI** | **LPCG** |
| **45** | **RFUSI-RMFG** | **RFUSI** | **45** | **LPCG-LIFG** | **LPCG** |
| **46** | **RFUSI-RMTG** | **RFUSI** | **46** | **LPCG-LITG** | **LPCG** |
| **47** | **RIFG-RFUSI** | **RIFG** | **47** | **LPCG-LMFG** | **LPCG** |
| **48** | **RIFG-RITG** | **RIFG** | **48** | **LPCG-LMTG** | **LPCG** |
| **49** | **RIFG-RMFG** | **RIFG** | **49** | **LPCG-LSFG** | **LPCG** |
| **50** | **RIFG-RMTG** | **RIFG** | **50** | **LSFG-LAG** | **LSFG** |
| **51** | **RITG-RFUSI** | **RITG** | **51** | **LSFG-LFUSI** | **LSFG** |
| **52** | **RITG-RIFG** | **RITG** | **52** | **LSFG-LIFG** | **LSFG** |
| **53** | **RITG-RMFG** | **RITG** | **53** | **LSFG-LITG** | **LSFG** |
| **54** | **RITG-RMTG** | **RITG** | **54** | **LSFG-LMFG** | **LSFG** |
| **55** | **RMFG-RFUSI** | **RMFG** | **55** | **LSFG-LMTG** | **LSFG** |
| **56** | **RMFG-RIFG** | **RMFG** | **56** | **LSFG-LPCG** | **LSFG** |
| **57** | **RMFG-RITG** | **RMFG** | **57** | **RAG-RFUSI** | **RAG** |
| **58** | **RMFG-RMTG** | **RMFG** | **58** | **RAG-RIFG** | **RAG** |
| **59** | **RMTG-RFUSI** | **RMTG** | **59** | **RAG-RMTG** | **RAG** |
| **60** | **RMTG-RIFG** | **RMTG** | **60** | **RFUSI-RAG** | **RFUSI** |
| **61** | **RMTG-RITG** | **RMTG** | **61** | **RFUSI-RIFG** | **RFUSI** |
| **62** | **RMTG-RMFG** | **RMTG** | **62** | **RFUSI-RMTG** | **RFUSI** |
| **63** | **LFUSI-RFUSI** | **LFUSI** | **63** | **RIFG-RAG** | **RIFG** |
| **64** | **LIFG-RIFG** | **LIFG** | **64** | **RIFG-RFUSI** | **RIFG** |
| **65** | **LITG-RITG** | **LITG** | **65** | **RIFG-RMTG** | **RIFG** |
| **66** | **LMFG-RMFG** | **LMFG** | **66** | **RMTG-RAG** | **RMTG** |
| **67** | **LMTG-RMTG** | **LMTG** | **67** | **RMTG-RFUSI** | **RMTG** |
| **68** | **RFUSI-LFUSI** | **RFUSI** | **68** | **RMTG-RIFG** | **RMTG** |
| **69** | **RIFG-LIFG** | **RIFG** | **69** | **LAG-RAG** | **LAG** |
| **70** | **RITG-LITG** | **RITG** | **70** | **LFUSI-RFUSI** | **LFUSI** |
| **71** | **RMFG-LMFG** | **RMFG** | **71** | **LIFG-RIFG** | **LIFG** |
| **72** | **RMTG-LMTG** | **RMTG** | **72** | **LMTG-RMTG** | **LMTG** |
|  |  |  | **73** | **RAG-LAG** | **RAG** |
|  |  |  | **74** | **RFUSI-LFUSI** | **RFUSI** |
|  |  |  | **75** | **RIFG-LIFG** | **RIFG** |
|  |  |  | **76** | **RMTG-LMTG** | **RMTG** |

**fMRI activation results for the untrained category.**

**Table 3: Following are the activated regions with T-values for picture naming task across patients for the post>pre rehabilitation untrained category (voxels active at *p* <0.001 uncorrected have threshold at T values between 3.10<T<4.80 while voxels active at *p* <0.05 FWE have threshold at T values T>4.80).**

| **Picture Naming** | **Patients: Untrained category** | | | | | |
| --- | --- | --- | --- | --- | --- | --- |
|  | Post > Pre | | | | | |
|  | #15 | #32 | #33 | #62 | #93 | #115 |
| **Anterior Left** |  | | | | | |
| Left Superior Frontal Gyrus | 3.56 | 4.20 | 3.33 |  | 4.06 | 3.64 |
| Left Middle Frontal Gyrus | 4.10 | 4.62 | 3.41 |  | 6.05 | 4.15 |
| Left Inferior Frontal Gyrus | 3.63 | 4.67 | 4.27 |  | 4.82 | 3.78 |
| Left Precentral Gyrus | 3.90 | 3.84 | 3.13 |  | 5.15 |  |
| **Anterior Right** |  | | | | | |
| Right Superior Frontal Gyrus | 3.92 | 5.05 | 3.69 |  | 5.67 | 3.38 |
| Right Middle Frontal Gyrus | 3.63 | 3.73 | 3.81 |  | 5.17 |  |
| Right Inferior Frontal Gyrus | 4.72 |  | 3.92 |  | 3.77 | 3.80 |
| Right Precentral Gyrus |  |  |  |  |  |  |
| **Posterior Left** |  | | | | | |
| Left Superior Temporal Gyrus | 5.76 | 4.09 | 3.64 |  | 4.07 | 3.16 |
| Left Middle Temporal Gyrus | 4.65 | 4.15 | 3.98 |  | 4.63 | 4.37 |
| Left Inferior Temporal Gyrus | 4.09 | 4.62 |  |  | 3.32 |  |
| Left Fusiform Gyrus | 4.90 | 3.39 | 3.31 |  |  | 3.33 |
| Left SupraMarginal Gyrus | 4.90 | 4.68 | 4.56 |  | 3.33 | 3.17 |
| Left Angular Gyrus | 4.06 | 3.85 | 4.90 |  | 3.14 | 3.17 |
| **Posterior Right** |  | | | | | |
| Right Superior Temporal Gyrus | 4.22 |  | 4.77 |  | 4.09 |  |
| Right Heschls Gyrus |  |  | 4.43 |  | 3.44 |  |
| Right Middle Temporal Gyrus | 3.84 | 3.51 | 4.36 |  | 4.34 |  |
| Right Inferior Temporal Gyrus | 4.28 | 3.48 |  |  |  |  |
| Right Fusiform Gyrus | 5.11 |  | 3.16 |  |  |  |
| Right SupraMarginal Gyrus | 4.32 | 3.22 | 3.56 |  | 4.39 |  |
| Right Angular Gyrus | 4.67 |  | 3.80 |  | 3.87 |  |

**Table 4. Following are the activated regions with T-values for semantic feature task across patients for the post>pre rehabilitation untrained category (voxels active at *p* <0.001 uncorrected have threshold at T values between 3.10<T<4.80 while voxels active at *p* <0.05 FWE have threshold at T values T>4.80).**

| **Semantic Feature** | **Patients: Untrained category** | | | | | |
| --- | --- | --- | --- | --- | --- | --- |
|  | Post > Pre | | | | | |
|  | #15 | #32 | #33 | #62 | #93 | #115 |
| **Anterior Left** |  | | | | | |
| Left Superior Frontal Gyrus | 8.16 | 4.12 | 3.90 |  |  | 4.38 |
| Left Middle Frontal Gyrus | 4.50 |  | 4.28 |  |  | 4.01 |
| Left Inferior Frontal Gyrus | 5.46 |  | 4.22 |  | 4.06 | 4.47 |
| Left Precentral Gyrus | 5.13 | 3.61 | 5.26 |  |  | 4.45 |
| **Anterior Right** |  | | | | | |
| Right Superior Frontal Gyrus | 6.64 | 5.05 | 5.29 |  | 3.25 | 3.93 |
| Right Middle Frontal Gyrus | 6.17 | 3.62 | 4.24 |  | 3.52 | 4.20 |
| Right Inferior Frontal Gyrus | 7.45 |  | 4.98 |  | 4.10 | 3.97 |
| Right Precentral Gyrus |  |  |  |  |  |  |
| **Posterior Left** |  | | | | | |
| Left Superior Temporal Gyrus | 5.93 |  | 4.36 |  | 4.00 | 3.20 |
| Left Middle Temporal Gyrus | 6.08 | 4.57 | 4.88 |  | 3.52 | 5.14 |
| Left Inferior Temporal Gyrus |  |  | 3.44 |  | 3.34 | 4.14 |
| Left Fusiform Gyrus | 3.19 | 3.48 | 4.11 |  |  | 4.30 |
| Left SupraMarginal Gyrus | 6.50 |  | 3.55 |  |  |  |
| Left Angular Gyrus | 5.33 | 3.18 | 3.99 |  |  | 4.65 |
| **Posterior Right** |  | | | | | |
| Right Superior Temporal Gyrus | 3.35 | 3.53 | 4.35 |  |  |  |
| Right Heschls Gyrus |  |  | 4.15 |  | 3.71 |  |
| Right Middle Temporal Gyrus | 3.75 | 4.64 | 4.27 |  | 3.30 |  |
| Right Inferior Temporal Gyrus |  | 3.22 | 3.96 |  |  | 3.32 |
| Right Fusiform Gyrus |  | 3.92 | 4.20 |  | 3.34 | 4.38 |
| Right SupraMarginal Gyrus |  | 3.60 | 5.94 |  |  |  |
| Right Angular Gyrus | 3.83 | 3.13 | 3.99 |  |  |  |

**Table 5: The supplementary table explains model space for each individual patient. Each patient has set of model space based of VOI's active pre and post for each task. Intrinsic connections (A-matrix) was setup for connections across all regions within hemisphere, while connections across homologous regions between hemisphere. The table contains Model#, B-input and C-input; B-input= effect of condition pictures on the connection; C-input= effect of connection pictures on the region. Bayesian Parameter Average was performed for models with same structure across pre and post scan.**

| Picture Naming | | | Semantic Feature | | |
| --- | --- | --- | --- | --- | --- |
| BUMA05 |  |  | BUMA05 |  |  |
| model# | B-Matrix | C-Matrix | model# | B-Matrix | C-Matrix |
| 1 | LAG-LIFG | LAG | 1 | RSFG-RMFG | RSFG |
| 2 | LAG-LPCG | LAG | 2 | RMFG-RSFG | RMFG |
| 3 | LIFG-LAG | LIFG | 3 | RMFG-RIFG | RMFG |
| 4 | LIFG-LPCG | LIFG | 4 | RIFG-RMFG | RIFG |
| 5 | LPCG-LAG | LPCG | 5 | RSFG-RIFG | RSFG |
| 6 | LPCG-LIFG | LPCG | 6 | RIFG-RSFG | RIFG |
| 7 | LAG-LIFG,LAG-LPCG | LAG | 7 | LIFG-RIFG | LIFG |
| 8 | LIFG-LAG,LIFG-LPCG | LIFG | 8 | RIFG-LIFG | RIFG |
| 9 | LPCG-LAG,LPCG-LIFG | LPCG | 9 | RSFG-RMFG | RSFG,RMFG |
|  |  |  | 10 | RMFG-RIFG | RMFG,RIFG |
|  |  |  | 11 | RSFG-RIFG | RSFG,RIFG |
|  |  |  | 12 | LIFG-RIFG | LIFG,RIFG |
|  |  |  | 13 | RSFG-RMFG,RMFG-RIFG | RSFG |
|  |  |  | 14 | RSFG-RMFG,RMFG-RIFG | RSFG,RMFG |
|  |  |  | 15 | RSFG-RMFG,RMFG-RIFG | RSFG,RMFG,RIFG |
|  |  |  | 16 | RIFG-RMFG,RMFG-RSFG | RIFG |
|  |  |  | 17 | RIFG-RMFG,RMFG-RSFG | RIFG,RMFG |
|  |  |  | 18 | RIFG-RMFG,RMFG-RSFG | RIFG,RMFG,RSFG |
|  |  |  | 19 | RSFG-RMFG,RMFG-RIFG | RSFG,LIFG |
|  |  |  | 20 | RSFG-RMFG,RMFG-RIFG | RSFG,RMFG,LIFG |
|  |  |  | 21 | RSFG-RMFG,RMFG-RIFG | RSFG,RMFG,RIFG |
|  |  |  | 22 | RIFG-RMFG,RMFG-RSFG | RIFG,LIFG |
|  |  |  | 23 | RIFG-RMFG,RMFG-RSFG | RIFG,RMFG,LIFG |
|  |  |  | 24 | RIFG-RMFG,RMFG-RSFG | RIFG,RMFG,RSFG |
|  |  |  | 25 | RSFG-RMFG | RSFG,LIFG |
|  |  |  | 26 | RMFG-RSFG | RMFG,LIFG |
|  |  |  | 27 | RMFG-RIFG | RMFG,LIFG |
|  |  |  | 28 | RIFG-RMFG | RIFG,LIFG |
|  |  |  | 29 | RSFG-RIFG | RSFG,LIFG |
|  |  |  | 30 | RIFG-RSFG | RIFG,LIFG |
|  |  |  | 31 | RIFG-LIFG | RIFG,LIFG |
| BUMA11 |  |  | BUMA11 |  |  |
| model# | B-Matrix | C-Matrix | model# | B-Matrix | C-Matrix |
| 1 | LIFG-RIFG,RIFG-LIFG | LIFG | 1 | LIFG-LMFG | LIFG |
| 2 | RIFG-LIFG | RIFG | 2 | LIFG-LMTG | LIFG |
| 3 | LIFG-LITG | LIFG | 3 | LIFG-LPCG | LIFG |
| 4 | LIFG-LPCG | LIFG | 4 | LIFG-LSFG | LIFG |
| 5 | LITG-LIFG | LITG | 5 | LMFG-LIFGRI | LMFG |
| 6 | LITG-LPCG | LITG | 6 | LMFG-LMTG | LMFG |
| 7 | LPCG-LIFG | LPCG | 7 | LMFG-LPCG | LMFG |
| 8 | LPCG-LITG | LPCG | 8 | LMFG-LSFG | LMFG |
| 9 | RIFG-RMFG | RIFG | 9 | LMTG-LIFG | LMTG |
| 10 | RMFG-RIFG | RMFG | 10 | LMTG-LMFG | LMTG |
| 11 | LIFG-LITG, LIFG-LPCG | LIFG | 11 | LMTG-LPCG | LMTG |
| 12 | LITG-LIFG,LITG-LPCG | LITG | 12 | LMTG-LSFG | LMTG |
| 13 | LPCG-LIFG,LPCG-LITG | LPCG | 13 | LPCG-LIFG | LPCG |
| 14 | RIFG-RMFG,RMFG-RIFG | RIFG,RMFG | 14 | LPCG-LMFG | LPCG |
| 15 | LIFG-RIFG,RIFG-LIFG | LIFG,RIFG | 15 | LPCG-LMTG | LPCG |
|  |  |  | 16 | LPCG-LSFG | LPCG |
|  |  |  | 17 | LSFG-LIFG | LSFG |
|  |  |  | 18 | LSFG-LMFG | LSFG |
|  |  |  | 19 | LSFG-LMTG | LSFG |
|  |  |  | 20 | LSFG-LPCG | LSFG |
|  |  |  | 21 | RIFG-RMFG | RIFG |
|  |  |  | 22 | RIFG-RAG | RIFG |
|  |  |  | 23 | RMFG-RIFG | RMFG |
|  |  |  | 24 | RMFG-RAG | RMFG |
|  |  |  | 25 | RAG-RIFG | RAG |
|  |  |  | 26 | RAG-RMFG | RAG |
|  |  |  | 27 | LIFG-RIFG | LIFG |
|  |  |  | 28 | RIFG-LIFG | RIFG |
|  |  |  | 29 | LMFG-RMFG | LMFG |
|  |  |  | 30 | RMFG-LMFG | RMFG |
|  |  |  | 31 | LIFG-RIFG,LMFG-RMFG | LIFG,LMFG |
|  |  |  | 32 | RIFG-LIFG,RMFG-LMFG | RIFG,RMFG |
|  |  |  | 33 | LIFG-RIFG,RMFG-LMFG | LIFG,RMFG |
|  |  |  | 34 | LMFG-RMFG,RIFG-LIFG | LMFG,RIFG |
|  |  |  | 35 | LIFG-LMFG,LIFG-LMTG,LIFG-LPCG,LIFG-LSFG | LIFG |
|  |  |  | 36 | LMFG-LIFG,LMFG-LMTG,LMFG-LPCG,LMFG-LSFG | LMFG |
|  |  |  | 37 | LMTG-LIFG,LMTG-LMFG,LMTG-LPCG,LMTG-LSFG | LMTG |
|  |  |  | 38 | LPCG-LIFG,LPCG-LMFG,LPCG-LMTG,LPCG-LSFG | LPCG |
|  |  |  | 39 | LSFG-LIFG,LSFG-LMFG,LSFG-LMTG,LSFG-LPCG | LSFG |
|  |  |  | 40 | RIFG-RMFG,RIFG-RAG | RIFG |
|  |  |  | 41 | RMFG-RIFG,RMFG-RAG | RMFG |
|  |  |  | 42 | RAG-RIFG,RAG-RMFG | RAG |
| BUMA15 |  |  | BUMA15 |  |  |
| model# | B-Matrix | C-Matrix | model# | B-Matrix | C-Matrix |
| 1 | LSFG-LIFG | LSFG |  | LIFG-RIFG | LIFG |
| 2 | LSFG-LAG | LSFG |  | RIFG-LIFG | RIFG |
| 3 | LIFG-LSFG | LIFG |  | LIFG-RIFG,RIFG-LIFG | LIFG,RIFG |
| 4 | LIFG-LAG | LIFG |  |  |  |
| 5 | LAG-LSFG | LAG |  |  |  |
| 6 | LAG-LIFG | LAG |  |  |  |
| 7 | RMFG-RIFG | RMFG |  |  |  |
| 8 | RIFG-RMFG | RIFG |  |  |  |
| 9 | LIFG-RIFG | LIFG |  |  |  |
| 10 | RIFG-LIFG | RIFG |  |  |  |
| 11 | LSFG-LIFG,RMFG-RIFG | LSFG, RMFG |  |  |  |
| 12 | LSFG-LIFG,RMFG-RIFG | LSFG, RMFG, LAG |  |  |  |
| 13 | LSFG-LIFG,RMFG-RIFG, LAG-LIFG | LSFG, RMFG, LAG |  |  |  |
| 14 | LSFG-LIFG,RMFG-RIFG, LIFG-RIFG | LSFG, RMFG, LIFG |  |  |  |
| 15 | LSFG-LIFG,RMFG-RIFG, LIFG-RIFG | LSFG, RMFG, LAG, LIFG |  |  |  |
| 16 | LSFG-LIFG,RMFG-RIFG, LAG-LIFG, LIFG-RIFG | LSFG, RMFG, LAG, LIFG |  |  |  |
| 17 | LIFG-LSFG,RIFG-RMFG | LIFG,RIFG |  |  |  |
| 18 | LIFG-LSFG,RIFG-RMFG | LIFG,RIFG, LAG |  |  |  |
| 19 | LIFG-LSFG,RIFG-RMFG, LAG-LIFG | LIFG,RIFG, LAG |  |  |  |
| 20 | LIFG-LSFG,RIFG-RMFG, LIFG-RIFG | LIFG,RIFG |  |  |  |
| 21 | LIFG-LSFG,RIFG-RMFG, LIFG-RIFG | LIFG,RIFG, LAG, |  |  |  |
| 22 | LIFG-LSFG,RIFG-RMFG,, LAG-LIFG, LIFG-RIFG | LIFG,RIFG, LAG, |  |  |  |
| 23 | LSFG-LIFG,RMFG-RIFG, RIFG-LIFG | LSFG, RMFG, RIFG |  |  |  |
| 24 | LSFG-LIFG,RMFG-RIFG, RIFG-LIFG | LSFG, RMFG, LAG, RIFG |  |  |  |
| 25 | LSFG-LIFG,RMFG-RIFG, LAG-LIFG, RIFG-LIFG | LSFG, RMFG, LAG, RIFG |  |  |  |
| 26 | LIFG-LSFG,RIFG-RMFG, RIFG-LIFG | LIFG,RIFG |  |  |  |
| 27 | LIFG-LSFG,RIFG-RMFG, RIFG-LIFG | LIFG,RIFG, LAG, |  |  |  |
| 28 | LIFG-LSFG,RIFG-RMFG,, LAG-LIFG, RIFG-LIFG | LIFG,RIFG, LAG, |  |  |  |
| BUMA32 |  |  | BUMA32 |  |  |
| model# | B-Matrix | C-Matrix | model# | B-Matrix | C-Matrix |
| 1 | LFUSI-LIFG | LFUSI | 1 | LIFG-LMFG | LIFG |
| 2 | LFUSI-LITG | LFUSI | 2 | LIFG-LMTG | LIFG |
| 3 | LIFG-LFUSI | LIFG | 3 | LMFG-LIFG | LMFG |
| 4 | LIFG-LITG | LIFG | 4 | LMFG-LMTG | LMFG |
| 5 | LITG-LFUSI | LITG | 5 | LMTG-LIFG | LMTG |
| 6 | LITG-LIFG | LITG | 6 | LMTG-LMFG | LMTG |
| 7 | RFUSI-RIFG | RFUSI | 7 | RIFG-RITG | RIFG |
| 8 | RFUSI-RITG | RFUSI | 8 | RIFG-RMFG | RIFG |
| 9 | RIFG-RFUS | RIFG | 9 | RIFG-RMTG | RIFG |
| 10 | RIFG-RITG | RIFG | 10 | RIFG-RSFG | RIFG |
| 11 | RITG-RFUSI | RITG | 11 | RITG-RIFG | RITG |
| 12 | RITG-RIFG | RITG | 12 | RITG-RMFG | RITG |
| 13 | LFUSI-RFUSI | LFUSI | 13 | RITG-RMTG | RITG |
| 14 | LIFG-RIFG | LIFG | 14 | RITG-RSFG | RITG |
| 15 | LITG-RITG | LITG | 15 | RMFG-RIFG | RMFG |
| 16 | RFUSI-LFUSI | RFUSI | 16 | RMFG-RITG | RMFG |
| 17 | RIFG-LIFG | RIFG | 17 | RMFG-RMTG | RMFG |
| 18 | RITG-LITG | RITG | 18 | RMFG-RSFG | RMFG |
| 19 | LFUSI-LIFG,LFUSI-LITG | LFUSI | 19 | RMTG-RIFG | RMTG |
| 20 | LIFG-LFUSI,LIFG-LITG | LIFG | 20 | RMTG-RITG | RMTG |
| 21 | LITG-LFUSI,LITG-LIFG | LITG | 21 | RMTG-RMFG | RMTG |
| 22 | RFUSI-RIFG,RFUSI-RITG | RFUSI | 22 | RMTG-RSFG | RMTG |
| 23 | RIFG-RFUSI,RIFG-RITG | RIFG | 23 | RSFG-RIFG | RSFG |
| 24 | RITG-RFUSI,RITG-RIFG | RITG | 24 | RSFG-RITG | RSFG |
| 25 | LFUSI-LIFG,LFUSI-RFUSI | LFUSI | 25 | RSFG-RMFG | RSFG |
| 26 | LFUSI-LITG,LFUSI-RFUSI | LFUSI | 26 | RSFG-RMTG | RSFG |
| 27 | LIFG-LFUSI,LIFG-RIFG | LIFG | 27 | LIFG-RIFG | LIFG |
| 28 | LIFG-LITG,LIFG-RIFG | LIFG | 28 | LMFG-RMFG | LMFG |
| 29 | LITG-LFUSI,LITG-RITG | LITG | 29 | LMTG-RMTG | LMTG |
| 30 | LITG-LIFG,LITG-RITG | LITG | 30 | RIFG-LIFG | RIFG |
| 31 | RFUSI-RIFG,RFUSI-LFUSI | RFUSI | 31 | RMFG-LMFG | RMFG |
| 32 | RFUSI-RITG,RFUSI-LFUSI | RIFG | 32 | RMTG-LMTG | RMTG |
| 33 | RIFG-RFUSI,RIFG-LIFG | RIFG | 33 | LIFG-LMFG,LIFG-RIFG | LIFG |
| 34 | RIFG-RITG,RIFG-LIFG | RIFG | 34 | LIFG-LMTG,LIFG-RIFG | LIFG |
| 35 | RITG-RFUSI,RITG-LITG | RITG | 35 | LMFG-LIFG,LMFG-RMFG | LMFG |
| 36 | RITG-RIFG,RITG-LITG | RITG | 36 | LMFG-LMTG,LMFG-RMFG | LMFG |
| 37 | LFUSI-RFUSI,RFUSI-LFUSI | LFUSI,RFUSI | 37 | LMTG-LIFG,LMTG-RMTG | LMTG |
| 38 | LIFG-RIFG,RIFG-LIFG | LIFG,RIFG | 38 | LMTG-LMFG,LMTG-RMTG | LMTG |
| 39 | LITG-RITG,RITG-LITG | LITG,RITG | 39 | RIFG-RITG,RIFG-LIFG | RIFG |
|  |  |  | 40 | RIFG-RMFG,RIFG-LIFG | RIFG |
|  |  |  | 41 | RIFG-RMTG,RIFG-LIFG | RIFG |
|  |  |  | 42 | RIFG-RSFG,RIFG-LIFG | RIFG |
|  |  |  | 43 | RITG-RIFG,RIFG-LIFG | RIFG, RITG |
|  |  |  | 44 | RITG-RMFG,RMFG-LMFG | RITG, RMFG |
|  |  |  | 45 | RITG-RMTG,RMTG-LMTG | RITG,RMTG |
|  |  |  | 46 | RMFG-RIFG,RMFG-LMFG | RMFG |
|  |  |  | 47 | RMFG-RITG,RMFG-LMFG | RMFG |
|  |  |  | 48 | RMFG-RMTG,RMFG-LMFG | RMFG |
|  |  |  | 49 | RMFG-RSFG,RMFG-LMFG | RMFG |
|  |  |  | 50 | RMTG-RIFG,RMTG-LMTG | RMTG |
|  |  |  | 51 | RMTG-RITG,RMTG-LMTG | RMTG |
|  |  |  | 52 | RMTG-RMFG,RMTG-LMTG | RMTG |
|  |  |  | 53 | RMTG-RSFG,RMTG-LMTG | RMTG |
|  |  |  | 54 | RSFG-RIFG,RIFG-LIFG | RSFG,RIFG |
|  |  |  | 55 | RSFG-RMFG,RMFG-LMFG | RSFG,RMFG |
|  |  |  | 56 | RSFG-RMTG,RMTG-LMTG | RSFG,RMTG |
|  |  |  | 57 | LIFG-RIFG,RIFG-LIFG | LIFG,RIFG |
|  |  |  | 58 | LMFG-RMFG,RMFG-LMFG | LMFG,RMFG |
|  |  |  | 59 | LMTG-RMTG,RMTG-LMTG | LMTG,RMTG |
| BUMA33 |  |  | BUMA33 |  |  |
| model# | B-Matrix | C-Matrix | model# | B-Matrix | C-Matrix |
| 1 | LMFG-LIFG | LMFG | 1 | LFUSI-LIFG | LFUSI |
| 2 | LIFG-LMFG | LIFG | 2 | LFUSI-LMFG | LFUSI |
| 3 | RMFG-RIFG | RMFG | 3 | LFUSI-LSFG | LFUSI |
| 4 | RMFG-RAG | RMFG | 4 | LIFG-LFUSI | LIFG |
| 5 | RIFG-RAG | RIFG | 5 | LIFG-LMFG | LIFG |
| 6 | RIFG-RMFG | RIFG | 6 | LIFG-LSFG | LIFG |
| 7 | RAG-RMFG | RAG | 7 | LMFG-LFUSI | LMFG |
| 8 | RAG-RIFG | RAG | 8 | LMFG-LIFG | LMFG |
| 9 | LMFG-RMFG | LMFG | 9 | LMFG-LSFG | LMFG |
| 10 | RMFG-LMFG | RMFG | 10 | LSFG-LFUSI | LSFG |
| 11 | LIFG-RIFG | LIFG | 11 | LSFG-LIFG | LSFG |
| 12 | RIFG-LIFG | RIFG | 12 | LSFG-LMFG | LSFG |
| 13 | LMFG-LIFG,LIFG-LMFG | LMFG,LIFG | 13 | RAG-RFUSI | RAG |
| 14 | RMFG-RIFG,RMFG-RAG | RMFG | 14 | RAG-RMTG | RAG |
| 15 | RIFG-RMFG,RIFG-RAG | RIFG | 15 | RFUSI-RAG | RFUSI |
| 16 | RAG-RMFG,RAG-RIFG | RAG | 16 | RFUSI-RMTG | RFUSI |
|  |  |  | 17 | RMTG-RAG | RMTG |
|  |  |  | 18 | RMTG-RFUSI | RMTG |
|  |  |  | 19 | LFUSI-RFUSI | LFUSI |
|  |  |  | 20 | RFUSI-LFUSI | RFUSI |
|  |  |  | 21 | LFUSI-LIFG,LFUSI-LMFG | LFUSI |
|  |  |  | 22 | LFUSI-LIFG,LFUSI-LSFG | LFUSI |
|  |  |  | 23 | LIFG-LFUSI,LIFG-LMFG | LIFG |
|  |  |  | 24 | LIFG-LFUSI,LIFG-LSFG | LIFG |
|  |  |  | 25 | LMFG-LFUSI,LMFG-LIFG | LMFG |
|  |  |  | 26 | LMFG-LFUSI,LMFG-LSFG | LMFG |
|  |  |  | 27 | LSFG-LFUSI,LSFG-LIFG | LSFG |
|  |  |  | 28 | LSFG-LFUSI,LSFG-LMFG | LSFG |
|  |  |  | 29 | LSFG-LIFG,LSFG-LMFG | LSFG |
|  |  |  | 30 | RAG-RFUSI,RAG-RMTG | RAG |
|  |  |  | 31 | RFUSI-RAG,RFUSI-RMTG | RFUSI |
|  |  |  | 32 | RMTG-RAG,RMTG-RFUSI | RMTG |
|  |  |  | 33 | LFUSI-LIFG,LFUSI-LMFG,LFUSI-LSFG | LFUSI |
|  |  |  | 34 | LIFG-LFUSI,LIFG-LMFG,LIFG-LSFG | LIFG |
|  |  |  | 35 | LMFG-LFUSI,LMFG-LIFG,LMFG-LSFG | LMFG |
|  |  |  | 36 | LSFG-LFUSI,LSFG-LIFG,LSFG-LMFG | LSFG |
|  |  |  | 37 | LFUSI-LIFG,LFUSI-RFUSI | LFUSI |
|  |  |  | 38 | LFUSI-LMFG,LFUSI-RFUSI | LFUSI |
|  |  |  | 39 | LFUSI-LSFG,LFUSI-RFUSI | LFUSI |
|  |  |  | 40 | LIFG-LFUSI,LFUSI-RFUSI | LIFG,LFUSI |
|  |  |  | 41 | LIFG-LMFG,LFUSI-RFUSI | LIFG,LFUSI |
|  |  |  | 42 | LIFG-LSFG,LFUSI-RFUSI | LIFG,LFUSI |
|  |  |  | 43 | LMFG-LFUSI,LFUSI-RFUSI | LMFG,LFUSI |
|  |  |  | 44 | LMFG-LIFG,LFUSI-RFUSI | LMFG,LFUSI |
|  |  |  | 45 | LMFG-LSFG,LFUSI-RFUSI | LMFG,LFUSI |
|  |  |  | 46 | LSFG-LFUSI,LFUSI-RFUSI | LSFG,LFUSI |
|  |  |  | 47 | LSFG-LIFG,LFUSI-RFUSI | LSFG,LFUSI |
|  |  |  | 48 | LSFG-LMFG,LFUSI-RFUSI | LSFG,LFUSI |
|  |  |  | 49 | RAG-RFUSI,RFUSI-LFUSI | RAG,RFUSI |
|  |  |  | 50 | RAG-RMTG,RFUSI-LFUSI | RAG,RFUSI |
|  |  |  | 51 | RFUSI-RAG,RFUSI-LFUSI | RFUSI |
|  |  |  | 52 | RFUSI-RMTG,RFUSI-LFUSI | RFUSI |
|  |  |  | 53 | RMTG-RFUSI,RFUSI-LFUSI | RMTG,RFUSI |
|  |  |  | 54 | LFUSI-LIFG,LFUSI-LMFG,LFUSI-RFUSI | LFUSI |
|  |  |  | 55 | LFUSI-LIFG,LFUSI-LSFG,LFUSI-RFUSI | LFUSI |
|  |  |  | 56 | LIFG-LFUSI,LIFG-LMFG,LFUSI-RFUSI | LIFG,LFUSI |
|  |  |  | 57 | LIFG-LFUSI,LIFG-LSFG,LFUSI-RFUSI | LIFG,LFUSI |
|  |  |  | 58 | LMFG-LFUSI,LMFG-LIFG,LFUSI-RFUSI | LMFG,LFUSI |
|  |  |  | 59 | LMFG-LFUSI,LMFG-LSFG,LFUSI-RFUSI | LMFG,LFUSI |
|  |  |  | 60 | LSFG-LFUSI,LSFG-LIFG,LFUSI-RFUSI | LSFG,LFUSI |
|  |  |  | 61 | LSFG-LFUSI,LSFG-LMFG,LFUSI-RFUSI | LSFG,LFUSI |
|  |  |  | 62 | RAG-RFUSI,RAG-RMTG,RFUSI-LFUSI | RAG,RFUSI |
|  |  |  | 63 | RFUSI-RAG,RFUSI-RMTG,RFUSI-LFUSI | RFUSI |
|  |  |  | 64 | RMTG-RAG,RMTG-RFUSI,RFUSI-LFUSI | RMTG,RFUSI |
|  |  |  | 65 | LFUSI-LIFG,LFUSI-LMFG,LFUSI-LSFG,LFUSI-RFUSI | LFUSI |
|  |  |  | 66 | LIFG-LFUSI,LIFG-LMFG,LIFG-LSFG,LFUSI-RFUSI | LIFG,LFUSI |
|  |  |  | 67 | LMFG-LFUSI,LMFG-LIFG,LMFG-LSFG,LFUSI-RFUSI | LMFG,LFUSI |
|  |  |  | 68 | LSFG-LFUSI,LSFG-LIFG,LSFG-LMFG,LFUSI-RFUSI | LSFG,LFUSI |
| BUMA93 |  |  | BUMA93 |  |  |
| model# | B-Matrix | C-Matrix | model# | B-Matrix | C-Matrix |
| 1 | LSFG-LIFG | LSFG | 1 | LIFG-RIFG | LIFG |
| 2 | LSFG-LPCG | LSFG | 2 | RIFG-LIFG | RIFG |
| 3 | LSFG-LITG | LSFG | 3 | LMTG-RMTG | LMTG |
| 4 | LIFG-LSFG | LIFG | 4 | RMTG-LMTG | RMTG |
| 5 | LIFG-LPCG | LIFG | 5 | LSFG-LIFG | LSFG |
| 6 | LIFG-LITG | LIFG | 6 | LSFG-LMTG | LSFG |
| 7 | LITG-RITG | LITG | 7 | LIFG-LSFG | LIFG |
| 8 | RITG-LITG | RITG | 8 | LIFG-LMTG | LIFG |
| 9 | RSTG-RITG | RSTG | 9 | LMTG-LSFG | LMTG |
| 10 | RSTG-RAG | RSTG | 10 | LMTG-LIFG | LMTG |
| 11 | RITG-RSTG | RITG | 11 | RIFG-RMTG | RIFG |
| 12 | RITG-RAG | RITG | 12 | RIFG-RAG | RIFG |
| 13 | RAG-RSTG | RAG | 13 | RMTG-RIFG | RMTG |
| 14 | RAG-RITG | RAG | 14 | RMTG-RAG | RMTG |
| 15 | RSTG-RITG,RSTG-RAG | RSTG | 15 | RAG-RIFG | RAG |
| 16 | RITG-RSTG,RITG-RAG | RITG | 16 | RAG-RMTG | RAG |
| 17 | RAG-RSTG,RAG-RITG | RAG | 17 | LSFG-LIFG,LSFG-LMTG | LSFG |
| 18 | LSFG-LIFG,LSFG-LPCG | LSFG | 18 | LIFG-LSFG,LIFG-LMTG | LIFG |
| 19 | LSFG-LIFG,LSFG-LITG | LSFG | 19 | LMTG-LSFG,LMTG-LIFG | LMTG |
| 20 | LSFG-LPCG,LSFG-LITG | LSFG | 20 | RIFG-RMTG,RIFG-RAG | RIFG |
| 21 | LSFG-LIFG,LSFG-LPCG,LSFG-LITG | LSFG | 21 | RMTG-RIFG,RMTG-RAG | RMTG |
| 22 | LIFG-LSFG,LIFG-LPCG | LIFG | 22 | RAG-RIFG,RAG-RMTG | RAG |
| 23 | LIFG-LSFG,LIFG-LITG | LIFG | 23 | LIFG-RIFG,LMTG-RMTG | LIFG,LMTG |
| 24 | LIFG-LPCG,LIFG-LITG | LIFG | 24 | RIFG-LIFG,RMTG-LMTG | RIFG,RMTG |
| 25 | LIFG-LSFG,LIFG-LPCG,LIFG-LITG | LIFG | 25 | LIFG-RIFG,RIFG-LIFG | LIFG,RIFG |
| 26 | LPCG-LSFG,LPCG-LIFG | LPCG | 26 | LMTG-RMTG,RMTG-LMTG | LMTG,RMTG |
| 27 | LPCG-LSFG,LPCG-LITG | LPCG |  |  |  |
| 28 | LPCG-LIFG,LPCG-LITG | LPCG |  |  |  |
| 29 | LPCG-LSFG,LPCG-LIFG,LPCG-LITG | LPCG |  |  |  |
| 30 | LITG-LSFG,LITG-LIFG | LITG |  |  |  |
| 31 | LITG-LSFG,LITG-LPCG | LITG |  |  |  |
| 32 | LITG-LIFG,LITG-LPCG | LITG |  |  |  |
| 33 | LITG-LSFG,LITG-LPCG,LITG-LIFG | LITG |  |  |  |
| 34 | LITG-LSFG,LITG-LIFG,LITG-RITG | LITG |  |  |  |
| 35 | LITG-LSFG,LITG-LPCG,LITG-RITG | LITG |  |  |  |
| 36 | LITG-LIFG,LITG-LPCG,LITG-RITG | LITG |  |  |  |
| 37 | LITG-LSFG,LITG-LPCG,LITG-LIFG,LITG-RITG | LITG |  |  |  |
| 38 | RITG-RSTG,RITG-RAG,RITG-LITG | RITG |  |  |  |
| BUMA115 |  |  | BUMA115 |  |  |
| model# | B-Matrix | C-Matrix | model# | B-Matrix | C-Matrix |
| 1 | LMFG-LFUSI | LMFG | 1 | LMFG-LIFG | LMFG |
| 2 | LMFG-LIFG | LMFG | 2 | LMFG-LITG | LMFG |
| 3 | LMFG-LPCG | LMFG | 3 | LMFG-LPCG | LMFG |
| 4 | LFUSI-LMFG | LFUSI | 4 | LIFG-LMFG | LIFG |
| 5 | LFUSI-LIFG | LFUSI | 5 | LIFG-LITG | LIFG |
| 6 | LFUSI-LPCG | LFUSI | 6 | LIFG-LPCG | LIFG |
| 7 | LIFG-LMFG | LIFG | 7 | LITG-LMFG | LITG |
| 8 | LIFG-LFUSI | LIFG | 8 | LITG-LIFG | LITG |
| 9 | LIFG-LPCG | LIFG | 9 | LITG-LPCG | LITG |
| 10 | LPCG-LMFG | LPCG | 10 | LPCG-LMFG | LPCG |
| 11 | LPCG-LFUSI | LPCG | 11 | LPCG-LIFG | LPCG |
| 12 | LPCG-LIFG | LPCG | 12 | LPCG-LITG | LPCG |
| 13 | RFUSI-RIFG | RFUSI | 13 | RAG-RIFG | RAG |
| 14 | RFUSI-RMFG | RFUSI | 14 | RAG-RMFG | RAG |
| 15 | RFUSI-RSTG | RFUSI | 15 | RAG-RMTG | RAG |
| 16 | RIFG-RFUSI | RIFG | 16 | RIFG-RAG | RIFG |
| 17 | RIFG-RMFG | RIFG | 17 | RIFG-RMFG | RIFG |
| 18 | RIFG-RSTG | RIFG | 18 | RIFG-RMTG | RIFG |
| 19 | RMFG-RFUSI | RMFG | 19 | RMFG-RAG | RMFG |
| 20 | RMFG-RIFG | RMFG | 20 | RMFG-RIFG | RMFG |
| 21 | RMFG-RSTG | RMFG | 21 | RMFG-RMTG | RMFG |
| 22 | RSTG-RFUSI | RSTG | 22 | RMTG-RAG | RMTG |
| 23 | RSTG-RIFG | RSTG | 23 | RMTG-RIFG | RMTG |
| 24 | RSTG-RMFG | RSTG | 24 | RMTG-RMFG | RMTG |
| 25 | LMFG-RMFG | LMFG | 25 | LMFG-RMFG | LMFG |
| 26 | LIFG-RIFG | LIFG | 26 | LIFG-RIFG | LIFG |
| 27 | LFUSI-RFUSI | LFUSI | 27 | RMFG-LMFG | RMFG |
| 28 | RFUSI-LFUSI | RFUSI | 28 | RIFG-LIFG | RIFG |
| 29 | RIFG-LIFG | RIFG | 29 | LMFG-LIFG,LMFG-LITG | LMFG |
| 30 | RMFG-LMFG | RMFG | 30 | LMFG-LIFG,LMFG-LPCG | LMFG |
| 31 | LMFG-LFUSI,LMFG-LIFG | LMFG | 31 | LMFG-LITG,LMFG-LPCG | LMFG |
| 32 | LMFG-LFUSI,LMFG-LPCG | LMFG | 32 | LIFG-LMFG,LIFG-LITG | LIFG |
| 33 | LMFG-LIFG,LMFG-LPCG | LMFG | 33 | LIFG-LMFG,LIFG-LPCG | LIFG |
| 34 | LFUSI-LMFG,LFUSI-LIFG | LFUSI | 34 | LIFG-LITG,LIFG-LPCG | LIFG |
| 35 | LFUSI-LMFG,LFUSI-LPCG | LFUSI | 35 | LITG-LMFG,LITG-LIFG | LITG |
| 36 | LFUSI-LIFG,LFUSI-LPCG | LFUSI | 36 | LITG-LMFG,LITG-LPCG | LITG |
| 37 | LIFG-LMFG,LIFG-LFUSI | LIFG | 37 | LITG-LIFG,LITG-LPCG | LITG |
| 38 | LIFG-LMFG,LIFG-LPCG | LIFG | 38 | LPCG-LMFG,LPCG-LIFG | LPCG |
| 39 | LIFG-LFUSI,LIFG-LPCG | LIFG | 39 | LPCG-LMFG,LPCG-LITG | LPCG |
| 40 | LPCG-LMFG,LPCG-LFUSI | LPCG | 40 | LPCG-LIFG,LPCG-LITG | LPCG |
| 41 | LPCG-LMFG,LPCG-LIFG | LPCG | 41 | RAG-RIFG,RAG-RMFG | RAG |
| 42 | LPCG-LFUSI,LPCG-LIFG | LPCG | 42 | RAG-RIFG,RAG-RMTG | RAG |
| 43 | LMFG-LFUSI,LMFG-LIFG,LMFG-RMFG | LMFG | 43 | RAG-RMFG,RAG-RMTG | RAG |
| 44 | LMFG-LFUSI,LMFG-LPCG,LMFG-RMFG | LMFG | 44 | RIFG-RAG,RIFG-RMFG | RIFG |
| 45 | LMFG-LIFG,LMFG-LPCG,LMFG-RMFG | LMFG | 45 | RIFG-RAG,RIFG-RMTG | RIFG |
| 46 | LFUSI-LMFG,LFUSI-LIFG,LFUSI-RFUSI | LFUSI | 46 | RIFG-RMFG,RIFG-RMTG | RIFG |
| 47 | LFUSI-LMFG,LFUSI-LPCG,LFUSI-RFUSI | LFUSI | 47 | RMFG-RAG,RMFG-RIFG | RMFG |
| 48 | LFUSI-LIFG,LFUSI-LPCG,LFUSI-RFUSI | LFUSI | 48 | RMFG-RAG,RMFG-RMTG | RMFG |
| 49 | LIFG-LMFG,LIFG-LFUSI.LIFG-RIFG | LIFG | 49 | RMFG-RIFG,RMFG-RMTG | RMFG |
| 50 | LIFG-LMFG,LIFG-LPCG,LIFG-RIFG | LIFG | 50 | RMTG-RAG,RMTG-RIFG | RMTG |
| 51 | LIFG-LFUSI,LIFG-LPCG,LIFG-RIFG | LIFG | 51 | RMTG-RAG,RMTG-RMFG | RMTG |
| 52 | LPCG-LMFG,LPCG-LFUSI | LPCG | 52 | RMTG-RIFG,RMTG-RMFG | RMTG |
| 53 | LPCG-LMFG,LPCG-LIFG | LPCG | 53 | LMFG-LIFG,LMFG-LITG,LMFG-LPCG | LMFG |
| 54 | LPCG-LFUSI,LPCG-LIFG | LPCG | 54 | LIFG-LMFG,LIFG-LITG,LIFG-LPCG | LIFG |
| 55 | LMFG-LFUSI,LMFG-LIFG,LMFG-LPCG | LMFG | 55 | LITG-LMFG,LITG-LIFG,LITG-LPCG | LITG |
| 56 | LFUSI-LMFG,LFUSI-LIFG,LFUSI-LPCG | LFUSI | 56 | LPCG-LMFG,LPCG-LIFG,LPCG-LITG | LPCG |
| 57 | LIFG-LMFG,LIFG-LFUSI,LIFG-LPCG | LIFG | 57 | LMFG-LIFG,LMFG-LITG,LMFG-RMFG | LMFG |
| 58 | LPCG-LMFG,LPCG-LFUSI,LPCG-LIFG | LPCG | 58 | LMFG-LIFG,LMFG-LPCG,LMFG-RMFG | LMFG |
| 59 | LMFG-RMFG,LIFG-RIFG,LFUSI-RFUSI | LMFG, LIFG, LFUSI | 59 | LMFG-LITG,LMFG-LPCG,LMFG-RMFG | LMFG |
| 60 | LMFG-RMFG,RMFG-LMFG | LMFG,RMFG | 60 | LIFG-LMFG,LIFG-LITG,LIFG-RIFG | LIFG |
| 61 | LIFG-RIFG,RIFG-LIFG | LIFG,RIFG | 61 | LIFG-LMFG,LIFG-LPCG,LIFG-RIFG | LIFG |
| 62 | LFUSI-RFUSI,RFUSI-LFUSI | LFUSI,RFUSI | 62 | LIFG-LITG,LIFG-LPCG,LIFG-RIFG | LIFG |
| 63 | RFUSI-RIFG,RFUSI-RMFG | RFUSI | 63 | RIFG-RAG,RIFG-RMFG,RIFG-LIFG | RIFG |
| 64 | RFUSI-RIFG,RFUSI-RSTG | RFUSI | 64 | RIFG-RAG,RIFG-RMTG,RIFG-LIFG | RIFG |
| 65 | RFUSI-RMFG,RFUSI-RSTG | RFUSI | 65 | RIFG-RMFG,RIFG-RMTG,RIFG-LIFG | RIFG |
| 66 | RIFG-RFUSI,RIFG-RMFG | RIFG | 66 | RMFG-RAG,RMFG-RIFG,RMFG-LMFG | RMFG |
| 67 | RIFG-RFUSI,RIFG-RSTG | RIFG | 67 | RMFG-RAG,RMFG-RMTG,RMFG-LMFG | RMFG |
| 68 | RIFG-RMFG,RIFG-RSTG | RIFG | 68 | RMFG-RIFG,RMFG-RMTG,RMFG-LMFG | RMFG |
| 69 | RMFG-RFUSI,RMFG-RIFG | RMFG | 69 | LMFG-RMFG,LIFG-RIFG | LMFG,LIFG |
| 70 | RMFG-RFUSI,RMFG-RSTG | RMFG | 70 | RMFG-LMFG,RIFG-LIFG | RMFG,RIFG |
| 71 | RMFG-RIFG,RMFG-RSTG | RMFG |  |  |  |
| 72 | RSTG-RFUSI,RSTG-RIFG | RSTG |  |  |  |
| 73 | RSTG-RFUSI,RSTG-RMFG | RSTG |  |  |  |
| 74 | RSTG-RIFG,RSTG-RMFG | RSTG |  |  |  |
| 75 | RFUSI-RIFG,RFUSI-RMFG,RFUSI-LFUSI | RFUSI |  |  |  |
| 76 | RFUSI-RIFG,RFUSI-RSTG,RFUSI-LFUSI | RFUSI |  |  |  |
| 77 | RFUSI-RMFG,RFUSI-RSTG,RFUSI-LFUSI | RFUSI |  |  |  |
| 78 | RIFG-RFUSI,RIFG-RMFG,RIFG-LIFG | RIFG |  |  |  |
| 79 | RIFG-RFUSI,RIFG-RSTG,,RIFG-LIFG | RIFG |  |  |  |
| 80 | RIFG-RMFG,RIFG-RSTG,,RIFG-LIFG | RIFG |  |  |  |
| 81 | RMFG-RFUSI,RMFG-RIFG,RMFG-LMFG | RMFG |  |  |  |
| 82 | RMFG-RFUSI,RMFG-RSTG,RMFG-LMFG | RMFG |  |  |  |
| 83 | RMFG-RIFG,RMFG-RSTG,RMFG-LMFG | RMFG |  |  |  |
| 84 | RSTG-RFUSI,RSTG-RIFG | RSTG |  |  |  |
| 85 | RSTG-RFUSI,RSTG-RMFG | RSTG |  |  |  |
| 86 | RSTG-RIFG,RSTG-RMFG | RSTG |  |  |  |
| 87 | RFUSI-RIFG,RFUSI-RMFG,RFUSI-RSTG | RFUSI |  |  |  |
| 88 | RIFG-RFUSI,RIFG-RMFG,RIFG-RSTG | RIFG |  |  |  |
| 89 | RMFG-RFUSI,RMFG-RIFG,RMFG-RSTG | RMFG |  |  |  |
| 90 | RSTG-RFUSI,RSTG-RIFG,RSTG-RMFG | RSTG |  |  |  |
| 91 | RFUSI-LFUSI,RIFG-LIFG,RMFG-LMFG | RFUSI |  |  |  |

**fMRI Connectivity Results: Additional analyses**

Individual repeated measures ANOVAs on T1 and T2 Ep.B and Ep.C values as dependent measures and participant as the independent variable were performed for the trained category for the picture naming and semantic feature tasks. There was a significant effect of patient (*F* (1, 99) = 81.5, *p* <.0001), rehabilitation (*F* (1, 99) = 79.8, *p* <.0001), and interaction between rehabilitation and patient (*F* (1, 99) = 68.5, *p* <.0001). Post-hoc LSD tests (all differences significant at least *p* < .001) on the interaction effect showed that #15, #05 and #11 had significantly different Ep.B values T1 relative to T2 and these patients were significantly different from the rest of the patients. For Ep.C (regions) for the picture naming task, there was a significant effect of patient (*F* (1, 32) = 21.1, *p* <.0001), rehabilitation (*F* (1, 32) = 29.0, *p* <.0001), and interaction between rehabilitation and patient (*F* (1, 32) = 12.09, *p* <.0001). Post-hoc LSD tests (all differences significant at least *p* < .0001) on the interaction effect again showed that #15 and #11 had significantly different Ep.B values pre-rehabilitation relative to T2 and these patients were significantly different from the rest of the patients.

For the semantic feature task, when examining Ep.B (connections), there was a significant effect of patient (*F* (1, 129) = 48.9, *p* <.0001), rehabilitation (*F* (1, 129) = 36.4, *p* <.0001), and interaction between rehabilitation and patient (*F* (1, 129) = 15.8, *p* <.0001). Post-hoc LSD tests (all differences significant at least p < .001) on the interaction effect showed that #15, #33, #115 and #11 had had significantly different Ep.B values T1 relative to T2 and were significantly different from other patients. For Ep.C (regions), there was a significant effect of patient only (*F* (1, 36) = 17.2, *p* <.0001), and interaction between rehabilitation and patient (*F* (1, 36) = 3.6, *p* <.001). Post-hoc LSD tests (all differences significant at least *p* < .05) on the interaction effect showed that #15, #33, #05 had had significantly different Ep.C values T1 relative to T2.
